# Tuning wettability of molten lithium via a chemical strategy for lithium metal anodes

**DOI:** 10.1038/s41467-019-12938-4

**Published:** 2019-10-30

**Authors:** Shu-Hua Wang, Junpei Yue, Wei Dong, Tong-Tong Zuo, Jin-Yi Li, Xiaolong Liu, Xu-Dong Zhang, Lin Liu, Ji-Lei Shi, Ya-Xia Yin, Yu-Guo Guo

**Affiliations:** 1 CAS Key Laboratory of Molecular Nanostructure and Nanotechnology, CAS Research/Education Center for Excellence in Molecular Sciences, Beijing National Laboratory for Molecular Sciences (BNLMS), Institute of Chemistry, Chinese Academy of Sciences (CAS), 100190 Beijing, China; 2 University of Chinese Academy of Sciences, 100049 Beijing, China

**Keywords:** Electrochemistry, Energy

## Abstract

Metallic lithium affords the highest theoretical capacity and lowest electrochemical potential and is viewed as a leading contender as an anode for high-energy-density rechargeable batteries. However, the poor wettability of molten lithium does not allow it to spread across the surface of lithiophobic substrates, hindering the production and application of this anode. Here we report a general chemical strategy to overcome this dilemma by reacting molten lithium with functional organic coatings or elemental additives. The Gibbs formation energy and newly formed chemical bonds are found to be the governing factor for the wetting behavior. As a result of the improved wettability, a series of ultrathin lithium of 10–20 μm thick is obtained together with impressive electrochemical performance in lithium metal batteries. These findings provide an overall guide for tuning the wettability of molten lithium and offer an affordable strategy for the large-scale production of ultrathin lithium, and could be further extended to other alkali metals, such as sodium and potassium.

## Abstract (web summary)

Molten lithium cannot spread onto the lithiophobic substrate, hindering the production and application of lithium metal anodes. Here, the authors show a general chemical strategy to tune the wettability by forming new chemical bonds through the reactions with various organic coatings or elemental additives.

## Introduction

The rapid development of renewable clean energy technologies and the affiliated energy storage demand drive the intensive on-going research on high-energy-density (HED) rechargeable batteries^1–8^. Lithium (Li) metal rechargeable batteries are promising HED devices because Li metal anodes offer high specific capacity (3860 mA h g^−1^) and low electrochemical potential (−3.04 V vs. standard hydrogen electrode)^7,9–17^. Ultrathin Li with proper capacity (e.g., 3–6 mA h cm^−2^, 15–30 μm) rather than commercial Li foils (~110 mA h cm^−2^, 550 μm) is important for matching with state-of-art cathodes^3,15–19^, which are highly desired in HED Li batteries and prelithiation technology for silicon anodes^20–22^. It is a challenge to prepare the ultrathin Li with a thickness of 15–30 μm by rolling commercially thick Li foil, considering the poor mechanical properties and sticky issues of metallic Li during the rolling process. Spreading molten Li on Cu current collectors might be a promising strategy to realize the large-scale and low-cost preparation of ultrathin Li^23–26^. Unfortunately, this method suffers from the poor wettability of molten Li on various substrates. As a result, tuning the wettability of molten Li is of great importance in the field of Li metal batteries^27–33^.

Fundamentally, the spreading of molten Li requires high lithiophilicity of the substrate^23,24,26,28,29,31,33^. Most metal current collectors (e.g., Cu and Ni foam) and carbon materials used in Li metal batteries are lithiophobic to molten Li; furthermore, molten Li presents a liquid sphere on these substrates^24,26,33^. Accordingly, these substrates cannot be directly used as current collectors to spread molten Li. Recently, applying a thin interlayer, such as Al_2_O_3_, Si, Mg, Ge, Sn, and ZnO, has been proven effective inconsiderably improving molten Li wettability^23–25,28,33^. Nano-scale-thick interlayers have been realized via atomic layer deposition or chemical vapor deposition^33^. However, these surface modification technologies under high vacuum are time-consuming and costly^24^. Thus, highly effective and practical methods for tuning the wettability of molten Li must be explored.

Here we report the improvement of wettability of molten Li through systematic interphase design. The method is applicable to various substrates, and several types of ultrathin Li anode with thickness of 10–40 μm can be successfully achieved. First, a series of organic functional coatings can perfectly solve the poor wettability of molten Li. Many elemental additives that can react with molten Li can tune the surface energy of molten Li and hence facilitate the homogenous spreading of metallic Li on various substrates. Key parameters, such as Δ*rG* and newly formed chemical bonds, are found to be the governing factor for the improved wettability. The successful application of ultrathin Li anodes in rechargeable batteries highlights the importance of our strategy to improve the wettability of molten Li and further develop Li metal batteries toward next-generation energy storage systems.

## Results

### Preparation of ultrathin Li by tuning wettability of molten Li

**Fig. 1:**
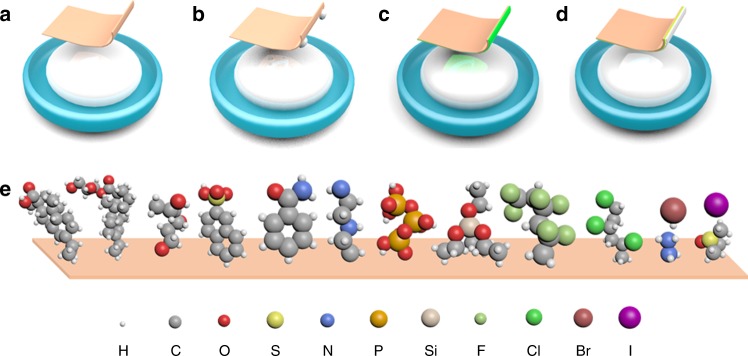
Schematic of an ultrathin Li layer formed onto lithiophobic substrates. Contact schematic of molten Li and the Cu substrate without (**a**, **b**) and with (**c**, **d**) the functional coating. **e** Molecular structures of various functional coatings

Constructing the appropriate interphase that facilitates the spread of molten Li onto the substrate is fundamental for ultrathin Li preparation (Fig. 1a–d). Herein, we developed a facile and low-cost chemical strategy for producing ultrathin Li anodes by introducing organic compounds with a functional group, such as –COOH, –OH, –SO_3_H, –NH_2_, –NH, –PO_4_, –Si-O, –F, –Cl, –Br, or –I, coated onto the substrates via a doctor-blade coating method (Fig. 1e). The reactions of the molten Li with organic compounds are the main driving force for the improved wettability. Therefore, designing the interphase reactions is the key step for the improved wettability.

**Fig. 2:**
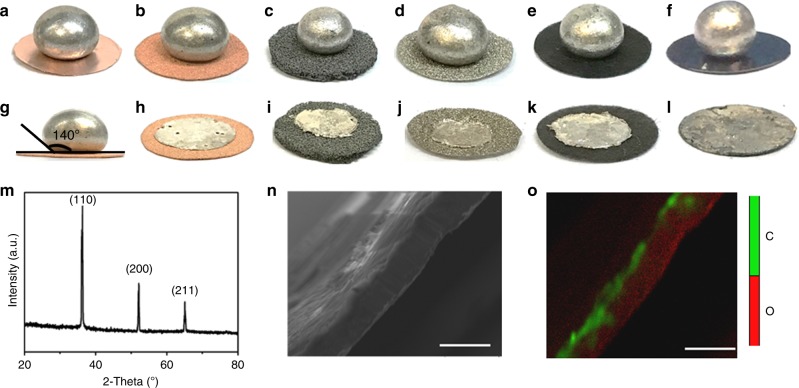
Wettability of molten Li onto various substrates and the prepared ultrathin Li layer. Molten Li shows a poor wettability on planar Cu (**a**), copper foam (**b**), iron foam (**c**), nickel foam (**d**), carbon fiber (**e**), and oxidized graphite (**f**). **g** The contact angle of planar Cu is 140°. With the organic coating of abietic resin, the surface wetting of molten Li onto various substrates is improved. Ultrathin Li is formed on copper foam (**h**), iron foam (**i**), nickel foam (**j**), carbon fiber (**k**), and oxidized graphite (**l**). **m** XRD of prepared ultrathin Li. **n** EPMA image of ultrathin Li. **o** Elemental distribution image of C and O on the ultrathin Li. Scale bars are 20 µm in **n**, **o**

The poor lithiophilicity of molten Li results in spherical Li beads rather than thin layers formed onto diverse substrates, including planar copper, foamed iron, foamed nickel, carbon fiber, and oxidized graphite, within the temperature range of 180–300 °C (Fig. 2a–f, Supplementary Movie 1). The contact angles of molten Li on these substrates were relatively large, (e.g., ~140 °C at a temperature of 250 °C for planar Cu; Fig. 2g). Abietic resin, a renewable and abundant acid in nature, is normally used as solder. Herein, it was used for the first time as a functional coating to modify the lithiophobic substrates. When abietic resin contacted with molten Li, a thermal decomposition reaction of abietic resin occurred (Supplementary Movie 2), and the resultant decomposition products immediately reacted with molten Li to form a new interphase. This reaction dramatically improved the wettability of molten Li and consequently formed ultrathin Li onto those lithiophobic substrates (Fig. 2h–l and Supplementary Movie 2–4). This process required <5 s for molten Li to spread across the substrates. Hence, it is easy to scale up for the continuous production of ultrathin Li. The result illustrates that the surface modification of Cu foil by abietic resin allows for good lithiophilicity with molten Li to generate ultrathin Li.

The formed ultrathin Li with thickness of ~12 μm (Fig. 2m, n) was characterized by X-ray diffraction (XRD), and all characteristic diffraction peaks could be identified as Li (Fig. 2m). The elemental analysis of electron microprobe analysis (EPMA) indicated that carbon was concentrated at the bottom surface of the Li layer (Fig. 2o). This carbon-enriched layer may mainly originate from the thermal decomposition of abietic resin. Interestingly, in addition to planar Cu substrate, ultrathin Li layers were also successfully prepared via abietic resin coatings on various substrates with different porous structures (e.g., Cu, carbon, and TiO_2_) with pore sizes ranging from nano to macro (Supplementary Figs. 1 and 2a). This result suggests that the coatings on substrates are the key point to govern the wettability of molten Li rather than pore structures or substrate constituents. Notably, when the concentration of abietic resin solution was further increased from 10 to 40 wt%, the molten Li could successfully infuse into the pores of carbon fiber felt (Supplementary Fig. 2b). This result indicates that the composite Li metal anode with three-dimensional (3D) conducting scaffold could be prepared easily by controlling the thickness of abietic resin.

Furthermore, we attempted a series of organic coatings containing functional groups of –COOH to determine the key factors for the improved wettability (Supplementary Table 1). Suitable melting points (24–275 °C) and boiling points (>150 °C) are the critical factors for the improved wettability. When the number of functional groups of –COOH was increased, dicarboxylic acids, such as oxalic acid, exhibited better performance for improving wettability than monocarboxylic acids, such as acetic acid or aminoacetic acid. It should be emphasized that the wettability of the organic coating on Cu substrate is commonly ignored, which is mainly determined by its physical properties and the selected solvents. Fortunately, the selected organic coating in a proper solvent could have good wettability on Cu substrates and thus facilitate molten Li spreading along substrates.

### Mechanism of improved wettability via abietic resin

As previously mentioned, diverse organic coatings have been used to improve the wettability of molten Li, and some empirical selection rules have been summarized. We sought to determine the dominating factors governing the improved wettability of molten Li. Thus, we conducted systematic studies to uncover the underlying mechanism.

**Fig. 3:**
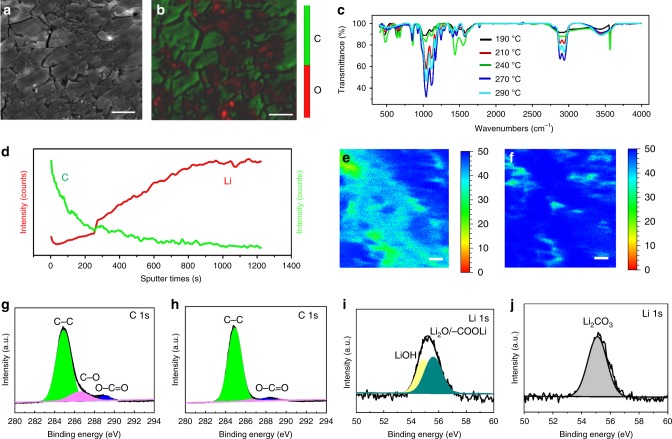
Mechanism of improved wettability on planar Cu by coating the substrate with abietic resin. **a** EPMA image of the bottom surface of ultrathin Li when it peeled off from the Cu substrate. **b** Elemental distribution image of O and C on the bottom surface. **c** Fourier infrared spectra of ultrathin Li on the bottom surface of the substrate. **d** Elemental distribution of Li^+^ and C^+^ probed via Tof-SIMS. **e** Elemental distribution map of Li^+^ on the bottom surface of ultrathin Li after etching 50 layers. **f** Elemental distribution map of Li^+^ on the bottom layer of ultrathin Li after etching 179 layers. **g** XPS spectra of C 1*s* for the abietic resin coating. XPS spectra of C 1*s* (**h**) and Li 1s (**i**) on the bottom surface of ultrathin Li layer when peeled off from the Cu substrate. **j** XPS spectra of Li 1*s* for the bottom surface of ultrathin Li after the carbon-enriched layer was removed by DOL. Scale bars are 20 µm in **a**, **b**, **e**, **f**

The morphology and element distribution of the top surface of ultrathin Li prepared from abietic resin were characterized by EPMA (Supplementary Fig. 3). The smooth top surface had similar compositions with commercial Li foil with carbon and oxygen contents of 7.32 and 92.68 wt%, respectively. This result indicates that the possible components of Li, Li_2_O, LiOH, and Li_2_CO_3_ existed at the top surface of ultrathin Li, and were derived from the inevitable slight oxidation of Li even in an argon-filled glove box. The Li layer was then peeled off from Cu substrate to expose the bottom surface of the ultrathin Li (Fig. 3a). EPMA results indicate that the interphase was composed of C (52.19 wt%) and O (47.81 wt%), as shown in Fig. 3b. Fourier transform infrared spectroscopy (FTIR; Fig. 3c, in combination with Supplementary Fig. 4a, b) confirms the existence of methyl and methylene groups (2900 cm^−1^), as well as LiOH at the bottom surface of Li layer. X-ray photoelectron spectroscopy (XPS) was used to identify the chemical state of the interphase. The C 1*s* spectrum could be deconvoluted into three peaks with binding energies of 284.8, 286.5, and 289 eV, which can be assigned to C–H and/or C–C, C–O, and –COO–. The peak intensity for C–O and –COO– was smaller compared with that in abietic resin (Fig. 3g), implying the decomposition of abietic resin. Two deconvoluted peaks in Li 1*s* spectrum could be assigned to LiOH (54.9 eV) and Li_2_O/–COOLi (55.6 eV). In combination with the above analysis, the compositions of carbon-enriched interphase can be deduced as follows: carbonaceous products, LiOH, and –COOLi/Li_2_O. Moreover, the chemical compositions of the interphase were similar over the temperature range of 180–300 °C (Fig. 3c), which allowed for a broad temperature range for abietic resin to improve wettability.

We then performed time-of-flight secondary ion mass spectrometry (Tof-SIMS) and XPS depth-profile study to determine the element distributions of the interphase from the bottom surface to bulk. The Tof-SIMS results in Fig. 3d–f show that Li^+^ content increased with the etching depth, whereas C^+^ content decreased. This finding implies that carbonaceous products gradually decreased with depth. The XPS depth profiles in Supplementary Figs. 5a, b further confirm these results. The compositions in the bulk were quite different from those on the bottom surface, where the peaks of Li 1*s* shifted to high binding energy (Supplementary Fig. 5b). Notably, the characteristic peaks of LiC_6_ and Li_2_C_2_ at 57.1 and 56.2 eV have been obtained through XPS simulation^34,35^, and Li_2_C_2_ and LiC_6_ can form according to the Li–C phase equilibria^36^. The Li_2_O/–COOLi, LiOH (Fig. 3i), LiC_6_, and Li_2_C_2_ (Supplementary Fig. 5b–d) on the interphase possibly originated from the reaction of molten Li with the decomposition products of abietic resin (trace water and pyrolyzed carbon). With increased etched depth, the contents of LiOH and Li_2_O/–COOLi significantly decreased, and those of LiC_6_ and Li_2_C_2_ increased. The different distributions of LiOH, Li_2_O/–COOLi, LiC_6_, and Li_2_C_2_ across the interphase reveal that the formed LiC_6_ and Li_2_C_2_ could suppress the diffusion of water vapor to the ultrathin Li. This result provides a new idea for the protection of Li against oxidation. In other words, these results indicate that the new interphase from abietic resin consisted of a mixture of LiOH, Li_2_O/–COOLi, LiC_6_, Li_2_C_2_, carboxy, methyl group, and methylene groups. The formation of the Li–O and Li–C bonds is the main driving force for the improved wettability of molten Li. For other carboxylic acids, such as citric acid, the mechanism for improved wettability is similar to that of abietic resin (Supplementary Fig. 6) because of the similar chemical properties from the carboxyl group.

It should be emphasized that the carbon-enriched interphase could be removed through washing with 1,3-dioxolane (DOL), offering a feasible procedure to eliminate the as-formed carbon-enriched interphase. The peak of Li_2_CO_3_ on Li metal at 55.1 eV was clearly detected at the bottom surface of ultrathin Li (Fig. 3j) after washing DOL. This peak might have originated from the oxidization of ultrathin Li during the washing process. Notably, the Li 1*s* binding energy of pure Li metal was 53.7 eV, but was difficult to be detected because of the inevitable oxidation of Li.

### Improved wettability via other organic coatings

Inspired by the chemical reaction between Li and abietic acid, we explored additional organic coatings from compounds containing –OH, –SO_3_H, –NH_2_, –NH, –PO_4_, –Si-O, –F, –Cl, –Br, or –I to improve the wettability of molten Li. Organic 2-naphthalenesulfonic acid with functional group of –SO_3_H was investigated for improved wettability. The appearance of LiOH peak reveals that H in –SO_3_H might play the same role with the H in –COOH (Supplementary Fig. 7). The peak at 54.5 eV could be attributed to LiOH or Li–O. Thus, the H in –SO_3_H might have been replaced by Li during the contact of Li with 2-naphthalenesulfonic acid, which was based on the displacement reaction between acids and metals. As the displacement reaction could be considered for improving the wettability of molten Li, tetraethyl orthosilicate (TEOS) was also coated onto planar Cu. TEOS could improve the wettability of molten Li. XPS peaks of SiO_2_ and SiO_*x*_–Li at 102.99 eV and 102.2 eV suggest the thermal decomposition of TEOS after the reaction (Supplementary Fig. 8a–c). The appearance of SiO_*x*_–Li might be due to the new phase SiO_*x*_ reacting with molten Li or molten Li directly displacing the ethyl group in TEOS. When the bottom surface of ultrathin Li was peeled off from the Cu substrate and washed by DOL, the characteristic peaks of the Li_2_CO_3_ (55.1 eV) and Li_2_O (55.6 eV) were observed as expected (Supplementary Fig. 8d).

Aside from the Li–O bond, Li–N bond could also be designed to improve wettability. The reaction mechanisms of molten Li with benzamide were confirmed with XPS analysis (Supplementary Fig. 9). After the contact of molten Li with benzamide, a peak from –N–Li was observed, suggesting that a displacement reaction occurred between Li and benzamide. Similarly, polyethyleneimine (PEI) was also used to improve wettability. The structure of PEI was not changed after the heat treatment (Supplementary Fig. 10), suggesting that no thermal decomposition occurred at 300 °C. Thus, the detected N–Li (398 eV) in Supplementary Fig. 11b confirms that the H in –N–H was directly displaced by Li.

Apart from the new bonds of Li–O or Li–N, other newly formed bonds, such as Li–F, Li–Cl, Li–Br, and Li–I, could also be designed. As expected, polyvinylidene fluoride (PVDF) coating successfully improved the wettability of molten Li, causing the ultrathin Li to spread onto the substrate. LiF could be detected from the Raman spectra when the ultrathin Li was peeled off from the substrate, which agrees with the commercial LiF powder (Supplementary Fig. 12a, b). Li–F (55.7 eV) and C–Li (55.2 eV) were also detected from XPS analysis (Supplementary Fig. 13), which indicates that the reaction of molten Li with PVDF is a complex chemical reaction. The change in FTIR spectra shows the changed structure from β-phase PVDF to α-phase PVDF at 300 °C (Supplementary Fig. 14). As a result, the observed C–Li at 55.2 eV originated from the displacement reaction between the molten Li and α-phase PVDF. However, it is a challenge to determine the specific substitution position of Li in PVDF. Beyond the new bonds of Li–F and Li–C, organic coatings in Supplementary Table 2 containing groups of –Br, –Cl, and –I were also designed to form new bonds containing Li–Br, Li–Cl, and Li–I, respectively. Hydrazine monohydrobromide, polyvinylchloride, and trimethylsulfoxonium iodide were coated onto the planar Cu substrate to improve the wettability of molten Li. The physical properties of these coatings are summarized in Supplementary Table 2. All of these formed new bonds contributed to improved wettability (Supplementary Table 3). The explored organic coatings exhibit great potential for tuning the wettability of molten Li because the coating process on substrates is quite simple and can be easily applied to a large-scale preparation. The prepared ultrathin Li with an area size of 6.5 cm × 4 cm is shown in Supplementary Fig. 15.

### Improved wettability based on elemental additives to molten Li

The surface tension of molten Li could be decreased to improve the wettability of molten Li on lithiophobic substrates. A direct-alloying method that involves adding carbon materials or tin into molten Li could improve the wettability of molten Li^24,37^. Other elements, such as indium (In) and magnesium (Mg) could also be successfully used to decrease the surface tension of molten Li at temperature ranges of 200–300 °C. Metal liquids with a decreased surface tension could spread onto Cu substrates (Supplementary Fig. 16). Thus, ultrathin Li alloy layers were successfully generated.

Typically, the surface tension of molten Li significantly decreased upon adding 10 wt% In into molten Li, and the molten Li–In could spread on the stainless steel with negligible contact angles after more than 10 min. When the amount of In was increased to 50 wt%, surface tension decreased, and molten Li spread on Cu substrates easily, suggesting that more In content is beneficial to weakening the inter-atomic force of Li. The as-prepared ultrathin Li_*x*_In_*y*_ anodes on planar Cu were characterized via EPMA (Supplementary Fig. 17a–d), identifying the existence of In in the prepared anodes. Only one XRD peak (36.26°) of Li_*x*_In_*y*_ was observed on planar Cu substrates (Supplementary Fig. 17e), because other peaks for Li_*x*_In_*y*_ were too weak to observe under extremely strong ground peaks from Cu substrate. When In content was increased to ~94 wt%, the LiIn alloy with the strongest peak of 37.28° was observed from XRD (Supplementary Fig. 17f). All of these results confirm the new bond formation, which decreased surface tension of molten Li, and improved wettability onto Cu substrates. Furthermore, other elemental additives [such as S (10 wt%), Se (50 wt%), Te (40 wt%), Bi (50 wt%), Pb (50 wt%), Ga (40 wt%), Cd (50 wt%), Hg (50 wt%), Pd (50 wt%), Sc (20 wt%), Y (40 wt%), Mg (28 wt%), Ca (40 wt%), Sr (50 wt%), and Ba (50 wt%)] were explored to decrease surface tension (Supplementary Table 4). After these alloying elemental additives were introduced into the molten Li, the surface tension of molten Li decreased, and the liquid metals could directly spread onto the planar Cu substrates, which agrees with a previous report of Sn^24^. These results suggest that adding alloying elements into molten Li is a general strategy to decrease the surface energy of the molten Li and improve wettability. As a result, the ultrathin Li composite could be prepared on lithiophobic substrates by decreasing the surface tension of liquid metals.

Li anodes with different thickness on Cu substrate were prepared via the PVDF coatings or via the elemental additives into the molten Li (Supplementary Fig. 18a–d). In comparison with the ultrathin Li prepared via organic coatings, ultrathin Li prepared by adding elemental additives could not be easily peeled off from the Cu substrate (Supplementary Fig. 18c, d). Ultrathin Li with thickness of ~40 μm was obtained (Supplementary Fig. 18d). The thickness of ultrathin Li on planar Cu could be regulated from several aspects, including temperature, composition of molten Li, and lithiophilic Pt coating on the planar Cu. The thickness of Li on the Cu substrate decreased with the increase in temperature from 250 to 500 °C (Supplementary Fig. 18e). In addition to the temperature of metal liquids, the composition of the metal liquids is a critical factor that determines the thickness of Li. In particles were added into the molten Li to form metal liquids at 300 °C. The thickness of Li on the Cu substrate increased with the In content. Furthermore, the thickness could also be tuned by coating lithiophilic Pt on the planar Cu. The different Li thickness might be attributed to the difference in the surface tension of the molten metal.

### Electrochemical testing of ultrathin Li

To evaluate the practicability of the resulting ultrathin Li, we utilized it as anodes in Li metal batteries. The areal capacity from 3.5 to 9 mA h cm^−2^ could be easily obtained by tuning the thickness of ultrathin Li (Supplementary Fig. 19a–c). The prepared ultrathin Li anode via PVDF exhibited a stable voltage profile with a small hysteresis (~40 mV), even after cycling for 500 h at the current density of 1 mA cm^−2^ with capacity of 2 mA h cm^−2^ (Supplementary Fig. 19d). The ultrathin Li prepared via PVDF coating on planar Cu current collectors was used as Li anodes, and commercial LiFePO_4_ was used as cathode (Supplementary Fig. 19e). The thickness of Li (e.g., the Li foil with thickness of 550 μm) could not guarantee the successful commercialization of Li metal batteries in the future. This condition is caused by the large excess of Li usage showing no advantages in improving energy density, but exhibiting a capacity degradation during cycling, which might be caused by the low Coulombic efficiency (CE) and uncontrolled Li dendrite growth. Furthermore, the ultrathin Li with thickness of 10–30 μm prepared via abietic resin coating was used as anodes. In comparison with the results in Supplementary Fig. 19f (Cu + commercial Li/LFP), the full cell exhibited a similar cycling behavior with a retention of ~88% after 250 cycles (Supplementary Fig. 20a). The capacity calculated based on the (cathode + anode) mass was 85.89 mA h g^−1^ for the ultrathin Li metal batteries (Supplementary Fig. 20a), which corresponds to an energy density of 292 Wh kg^−1^. We achieved a low energy density of 38 Wh kg^−1^ by increasing the Li:LFP mass ratio to 11.53:1 (with commercial Li metal of 550 μm as anode). With the increased CEs and suppressed dendrite growth in Li metal batteries, an increased energy density could be achieved by further reducing the Li/LFP mass ratio. Supplementary Figure 20b displays the cycling behavior of the full cell (Li–In/LiFePO_4_). Supplementary Figure 20d compares the voltage profiles of the LFP/Li cells with different anodes. The specific capacities of the two cells were similar with each other, that is, 135 mA h g^−1^ at 0.5 C for the Li–Mg anodes, and 140 mA h g^−1^ at 0.5 C for the Li–In anodes. The similar electrochemical performance might be due to the Li capacity being excessive for cathode capacity (Supplementary Table 5). The ultrathin Li could also be applied in other HED batteries with a Li metal anode and Ni-rich Li(Ni_*x*_Co_*y*_Mn_1 − *x* − *y*_)O_2_ cathode, possibly providing new perspectives for the development of high-performance Li metal batteries^38,39^.

In addition to the aforementioned full battery performance, we further investigated the plating behavior and electrochemical properties of ultrathin composite anodes prepared by elemental additives. With planar Cu as working electrodes, ultrathin Li_*x*_In_*y*_ anode (50 wt% In) deposited onto planar Cu substrate showed an overpotential of ~100 mV at a current density of 1 mA cm^−2^. For commercial Li anode, the value reached up to 150 mV (Supplementary Fig. 21a, b). In addition to the low overpotential, the morphology of Li_*x*_In_*y*_ anode was highly porous after the stripping of Li (Supplementary Fig. 21d), indicating a dealloying reaction during the stripping process (Li was extracted from Li_*x*_In_*y*_). Moreover, Li–Mg anodes prepared via adding 28 wt% Mg into molten Li showed not only compact electrodeposition morphologies (Supplementary Fig. 22a, c) but also low charge transfer resistance and a significant increase in cycling stability (Supplementary Fig. 23a, d), which are properties in contrast with those of the Li anode. Other elements (e.g., Hg, Ba, Sr, Y, Ga, Pd, Bi, S, Se, and Te) were used as additives to prepare ultrathin anodes, and the resultant anodes exhibited a remarkably compact and uniform electrodeposition during Li deposition (Supplementary Fig. 24). Thus, suggesting that the ultrathin anodes are promising to be obtained by tuning the wettability of molten Li via elemental additives.

Similar to molten Li, molten Na or K showed poor wettability onto various substrates even though these alkali metals could be used as the promising metal anodes for rechargeable batteries. On the basis of the aforementioned strategies for molten Li, molten Na was spread on lactic acid-coated Cu substrates. Subsequently, an ultrathin Na layer <1 μm was successfully produced (Supplementary Fig. 25a). The elemental distribution of C, O, and Na at the bottom surface suggests that molten Na could react with the thermal decomposition products of the organic coating and that the reaction has formed a new interface, thereby improving the wettability of molten Na (Supplementary Fig. 25b–g). These results suggest the great versatility of our wetting strategy in generating various ultrathin metal anodes and promoting its practical applications in next-generation rechargeable HED batteries.

## Discussion

**Fig. 4:**
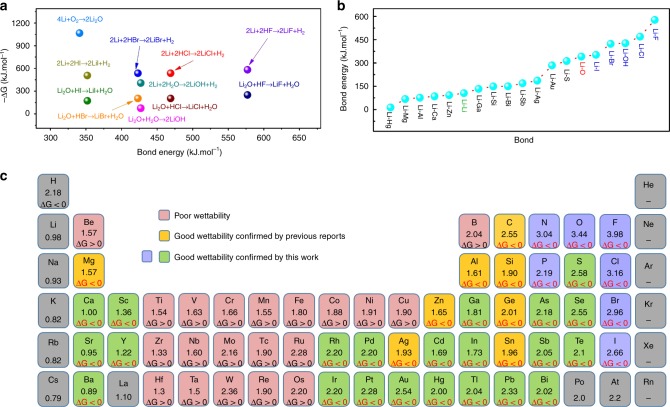
Common characteristics of wettability onto various lithiophobic substrates. **a** Δ_r_*G* for some feasible reactions at 250 °C to improve the wettability. **b** Bond energies of the newly formed bonds. **c** Electronegativities of various elements in the periodic table and Δ_r_*G* of elements or compounds reacted with the molten Li. The elements in green and blue colors represent the elements could react with molten Li at the temperature of 180–300 °C, and form new chemical bonds, which is responsible for the improved wettability

To better understand the key parameters governing the wettability, we summarized the related compounds and elements that could react with molten Li. As indicated by the aforementioned results, the improved wettability can be mainly attributed to the new chemical bonds formed as a result of the reaction between molten Li and lithiophilic substances. Given that the Δ_r_*G* gives the spontaneity of a reaction, from the thermodynamic viewpoint the negative values of Δ_r_*G* for those reactions were closely related to the improved wettability. Δ_r_*G* was calculated at a fixed temperature, as shown as follows^40^:

1 $$\Delta _{\mathrm{r}}G = \Delta _{\mathrm{r}}H_{298.15\,{\mathrm{K}}} - T\Delta _{\mathrm{r}}S_{298.15\,{\mathrm{K}}},$$

where Δ_r_*G* is the Gibbs formation energy for the reaction, Δ_r_*H*_298.15 K_ is the enthalpy change of the reaction at 298.15 K, Δ_r_*S*_298.15 K_ is the entropy change of the reaction at 298.15 K, and *T* is the reaction temperature. The temperature was set to 523.15 K. Δ_r_*G* for some feasible reactions at 250 °C to improve the wettability is shown in Fig. 4a and Supplementary Table 3. According to Eq. (1), the value of Δ_r_G could be significantly affected by temperature. For example, lithiophobic Cu substrates could be lithophilic when the temperature was increased to ~427 °C according to the Cu–Li phase diagram^41^.

Typically, the negative value of Δ_r_*G* for a specific reaction indicates that the newly formed chemical bond could be formed, which improves the wettability. The Δ_r_*G* of the reaction of Li and H_2_O (−407.7 kJ mol^−1^) was lower than that of Li_2_O and H_2_O (−74.6 kJ mol^−1^; Supplementary Table 3), indicating that the reaction is more favorable for Li and H_2_O than for Li_2_O and H_2_O. Moreover, the reactions of Li_2_O with alloying elements, such as Mg, Si, and Sn, could not occur, whereas the reaction of Li with these alloying elements (Δ_r_*G* <0) could occur at 250 °C (Supplementary Table 3). As a result, the oxidation of Li is unfavorable for the wettability of molten Li. Based on this principle, the heating temperature should be controlled, and the oxides of molten Li should be removed as much as possible. The formation of new related bonds, such as Li–X, which are responsible for improved wettability, is summarized in Fig. 4b. Notably, elements in the periodic table with red color (i.e., Ti, Fe, and Cu) could not react with the molten Li at the temperature range of 180–300 °C. Thus, the wettability of molten Li on those substrates is poor (Fig. 4c). On the contrary, lithiophilic coatings with functional groups containing –N, –P, –F, –Cl, –Br, or –I in blue color could react with molten Li, leading to new bond formation and thus improved wettability. Element additives in green color, such as In and Mg, could also be used to improve the wettability of molten Li. The summarized lithiophilic substances in the periodic table are potentially helpful in understanding the wetting phenomenon of molten alkali metals.

**Fig. 5:**
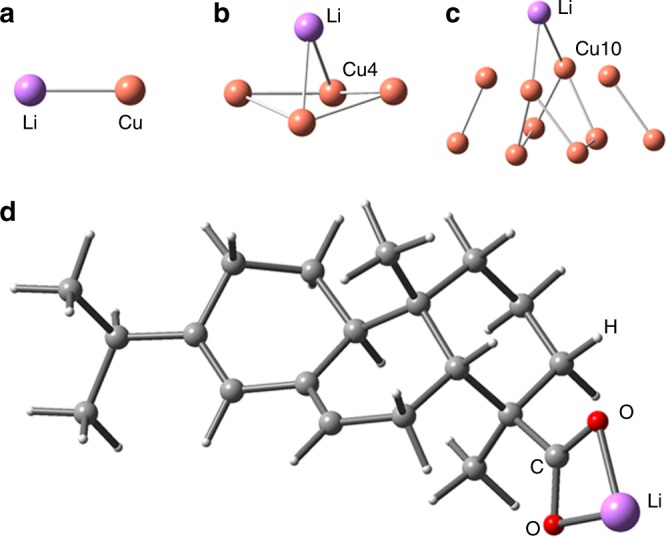
Theoretical calculation of the newly formed ionic bonds based on chemisorption. **a**–**c** The newly formed ionic bond between Li and Cu clusters. **d** The newly formed ionic bond between Li and abietic acid

In addition to Δ_r_*G*, the ionic bonding between Li and abietic acid was considered through the theoretical calculations based on chemisorption. The newly formed ionic bond between Cu clusters (Cu, Cu4, and Cu10) and Li was shown in Fig. 5a–c. The adsorption energies of Li on Cu, Cu4, and Cu10 clusters were −150.40, −123.81, and −94.28 kJ mol^−1^, respectively. The adsorption energy of Li on Cu decreased with the increase of cluster size. Furthermore, the adsorption of Li atoms on the surface of Cu would be further reduced in the actual system. The chemisorption energy of Li on abietic acid was −426.45 kJ mol^−1^, which is higher than that of Li on Cu clusters (i.e., Cu, Cu4, Cu10). From this point of view, the newly formed ionic bond between Li and abietic acid (Fig. 5d) was stronger than that of Li and Cu. Hence, the newly formed chemical bonds might be responsible for improved wettability.

In conclusion, we have developed a feasible chemical strategy for tuning the wettability of molten Li and successfully prepared a series of ultrathin Li anodes with the assistance of organic coatings with –COOH, –OH, –SO_3_H, –NH_2_, –NH-,–Si-O, –F, –Cl, –Br, –I, or elements including Mg, In, Ca, Sr, Ba, Sc, Y, Rh, Ir, Pd, Pt, Au, Cd, Hg, Ga, Tl, Ge, Pb, As, Sb, Bi, S, Se, and Te. For this newly developed chemical strategy, the mechanisms of improved wettability of molten Li are proposed. Negative values of Δ_r_*G* for the reactions between molten Li and lithiophilic substances and the newly formed bonds are regarded as characteristics that govern improved wettability. Furthermore, the chemisorption energy of Li on abietic acid is higher than that of Li on Cu cluster. Hence, the newly formed strong chemical bonds might be responsible for the improved wettability. As a result, ultrathin Li with the desired thickness could be easily prepared based on the newly developed, highly efficient, and toolkit-like chemical strategy, which could be successfully applied in HED batteries in the future.

## Methods

### Preparation of the abietic resin coating

The doctor-blade coating was adopted to form lithiophilic layers onto various lithiophobic substrates. A series of solutions were prepared. Abietic resin was dissolved in ethanol at a concentration of 5 wt%. Cu foil and other substrates were coated with the organic coating with thickness of ~200 μm (solvent containing). The solvent was fully volatilized before transferring to an argon-filled glove box with an oxygen and moisture <0.1 p.p.m. The thickness of the organic coating was ~500 nm after the volatilization of solvent. The preparation of other organic coatings was similar to the abietic resin.

### Preparation of the ultrathin Li layer via organic coatings

The commercialized Li was heated to 300 °C for 10 min. The gray oxides formed on molten Li at high temperatures even with the oxygen <0.1 p.p.m. The gray oxide layer was removed before the substrates contacted with the molten Li. Ultrathin Li layer could not be formed onto substrates without organic coatings at 180–300 °C. However, the ultrathin Li layer could be observed when substrates were coated with an organic coating such as abietic resin.

### Preparation of the ultrathin Li layer via decreasing surface tension of molten Li

Powders of In was added into molten Li at 300 °C with a weight ratio of 5, 10, 20, and 50 wt%, and In was distributed in the composites as homogeneous as possible. The new interphases were formed through a combination reaction. The surface tension of Li–In (5 wt% In) was not changed visibly. When the content of indium increases to 10 wt%, the surface energy could be tuned obviously. When the surface tension of molten Li was decreased, molten Li could spread onto the lithiophobic substrates such as planar Cu, forming ultrathin Li. Other elemental additives, such as S (10 wt%), Se (50 wt%), Te (40 wt%), Bi (50 wt%), Pb (50 wt%), Ga (40 wt%), Cd (50 wt%), Hg (50 wt%), Pd (50 wt%), Sc (20 wt%), Y (40 wt%), Mg (28 wt%), Ca (40 wt%), Sr (50 wt%), and Ba (50 wt%), were also investigated as additives to molten Li with the same procedure to tune the surface energy of molten Li.

## Supplementary information

Supplementary Information

Description of Additional Supplementary Files

Supplementary Movie 1

Supplementary Movie 2

Supplementary Movie 3

Supplementary Movie 4

## Data Availability

All data needed to evaluate the conclusions in the paper are present in the paper and/or the Supplementary Materials. The data that support the results within this paper are available from the corresponding author upon reasonable request.
