# Supplementary material for: Tuning wettability of molten lithium via a chemical strategy for lithium metal anodes
Source: Nat Commun. 2019 Oct 30;10:4930. doi: 10.1038/s41467-019-12938-4 (PMC6821877; doi:10.1038/s41467-019-12938-4)
Supplement: Supplementary file 1 — Supplementary Information [file 41467_2019_12938_MOESM1_ESM.pdf]

Supplementary information

Tuning wettability of molten lithium via a chemical strategy for lithium metal anode

Wang et al.

Supplementary Figures

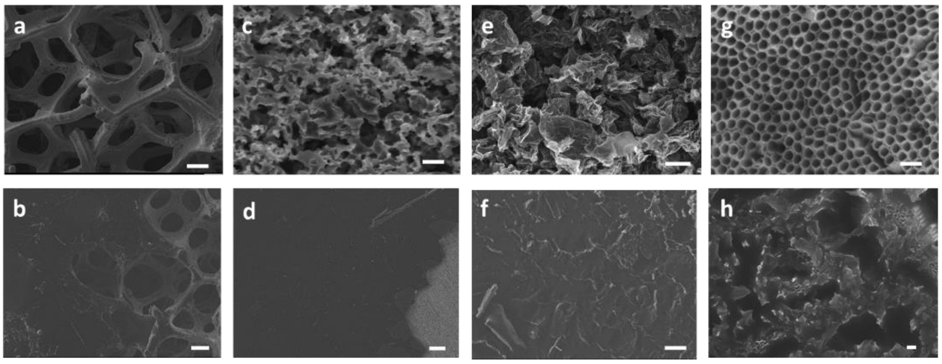

Supplementary Figure 1 | Characterization of the ultrathin Li on various substrates with different pore diameters. **a**, SEM image of the Cu foam. **b**, SEM image of ultrathin Li spreading on Cu foam. **c**, SEM image of the porous Cu substrate with a pore diameter of several micrometers. **d**, SEM image of ultrathin Li spreading on porous Cu. **e**, SEM image of porous graphene oxide. **f**, SEM image of ultrathin Li spreading on porous graphene oxide. **g**, SEM image of nano TiO_2 substrate. **h**, SEM image of ultrathin Li spreading on TiO_2 substrate. Scale bars for a and b are 100 μm; scale bar for c is 3 μm; scale bars for d,e, and f are 50 μm; scale bars for g and h are 200 nm.

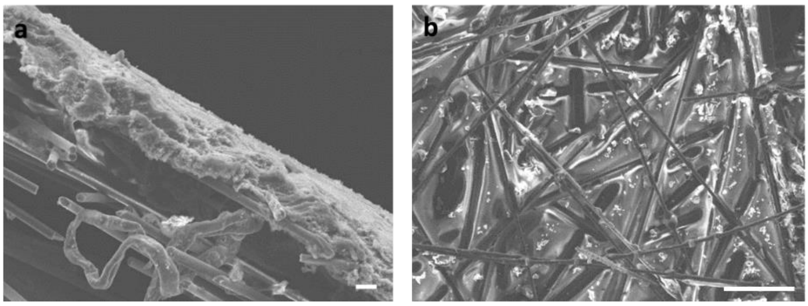

Supplementary Figure 2 | SEM images of Li formed on porous carbon substrates. **a**, SEM image of ultrathin Li layer spreading onto the carbon substrate when the substrate was coated 5 wt% abietic acid. **b**, SEM image of Li infusion into the pores with increasing the concentration of abietic acid solution to 40 wt%. Compared with planar substrates, porous substrates present higher surface area, which needs more abietic resin to achieve similar coverage of surface with the one on planar substrates. Increasing the concentration of abietic resin ethanol solution to 40 wt% will cause adequate abietic resin in the pores and thus facilitate more molten Li to permeate into the pores than that of 5 wt%. This might be the reason for 40 wt% abietic resin solution is needed for the porous substrate when the molten Li infused into the pores. Scale bar for a is 10 μm, for b is 100 μm.

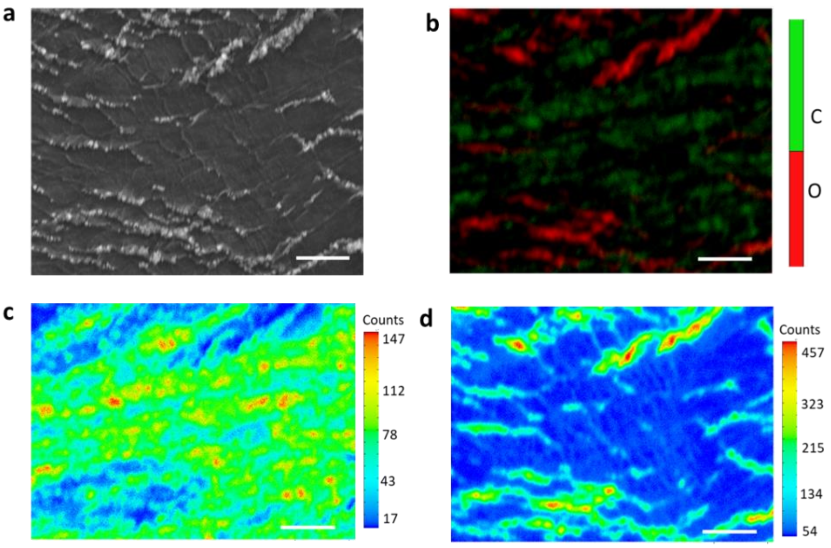

e

No.	Element	Line	Crystal	Peak WL(A)	K-ratio	Mass%	Mol%
1	C	Ka	PbST/CH2	44.5601	0.07324	7.32	9.52
2	O	Ka	RAP/CH1	23.5912	0.92676	92.68	90.48
						100.00	

Supplementary Figure 3 | EPMA of top surface of ultrathin Li. **a**, Surface morphology. **b**, Distribution of C and O on the surface. **c**, C distribution. **d**, O distribution. **e**, Element content table for C and O. Scale bars are 20 μm in a-d.

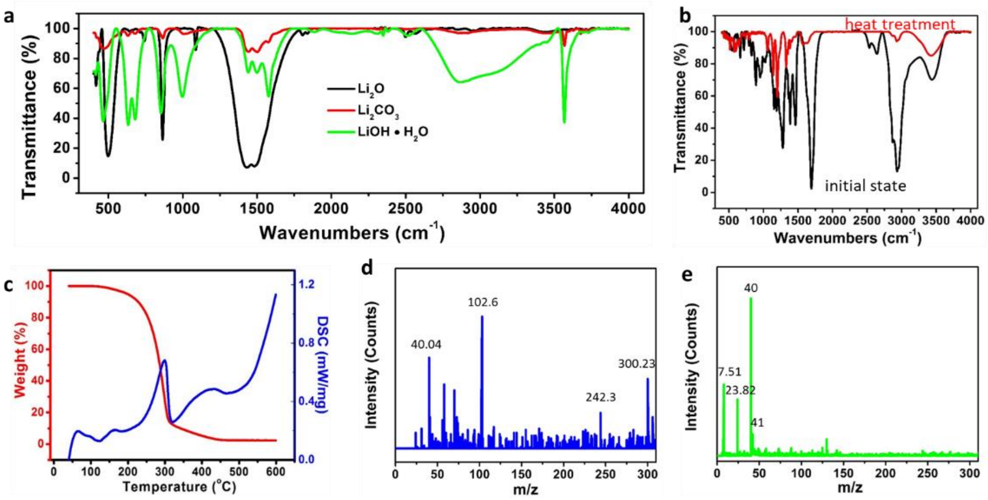

Supplementary Figure 4 | Mechanism analysis of abietic resin as the lithiophilic coating to improve wettability. **a**, IR of commercial Li_2O, Li_2CO_3, and LiOH. **b**, IR for abietic acid before and after the heat-treatment for 5 seconds at 250 °C. **c**, Thermogravimetric **(**TG) curve of abietic acid. **d** and **e**, Mass spectrum of abietic acid before (d) and after the heat treatment (e).

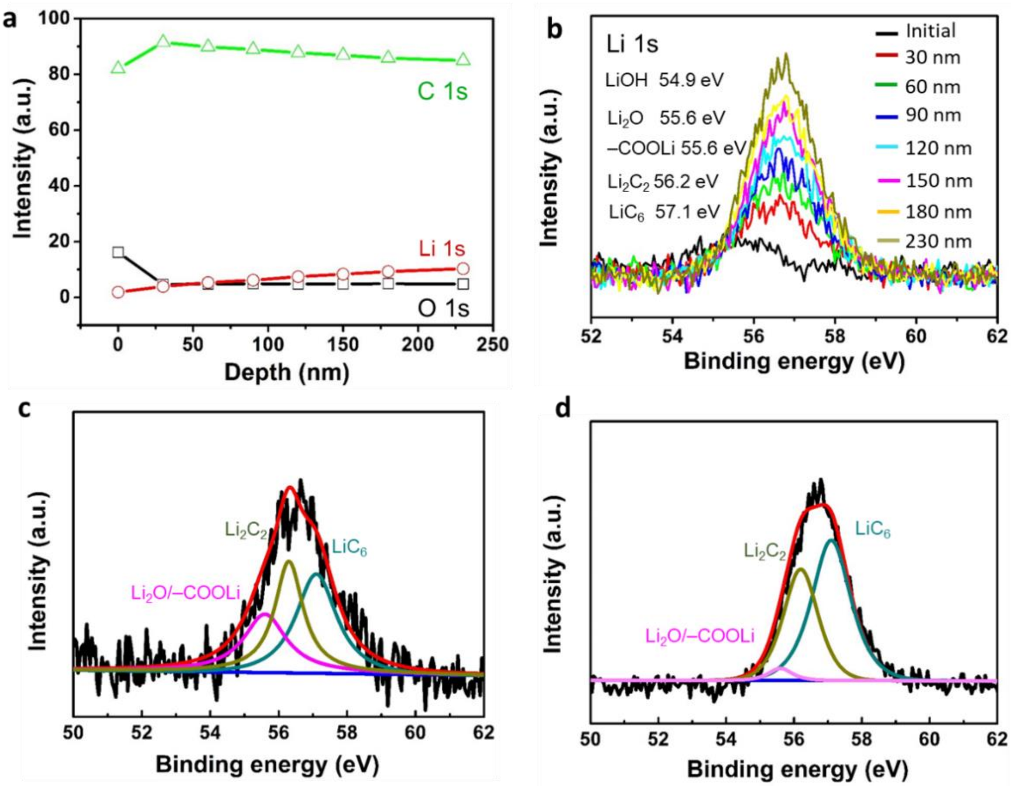

Supplementary Figure 5 | XPS depth profile analysis of the new interphase when abietic resin was used as the coating. a, Concentration-depth profile of Li 1s, O 1s, and C 1s. As we can see, Li content slightly increases while C content slightly decreases with etching depth. This can be explained by Li gradient diffusion into the interphase. b, Li 1s spectra for the original surface and after etching 30, 60, 90, 120, 150, 180, 230 nm by Ar^+ sputtering. It can be observed that the binding energy of Li 1s slightly increases with etching depth, indicating the change of the species of Li from LiOH to Li_2C_2 and LiC_6. **c** and **d**, Analysis of Li 1s XPS spectra after 30 nm and 230 nm etching, respectively.

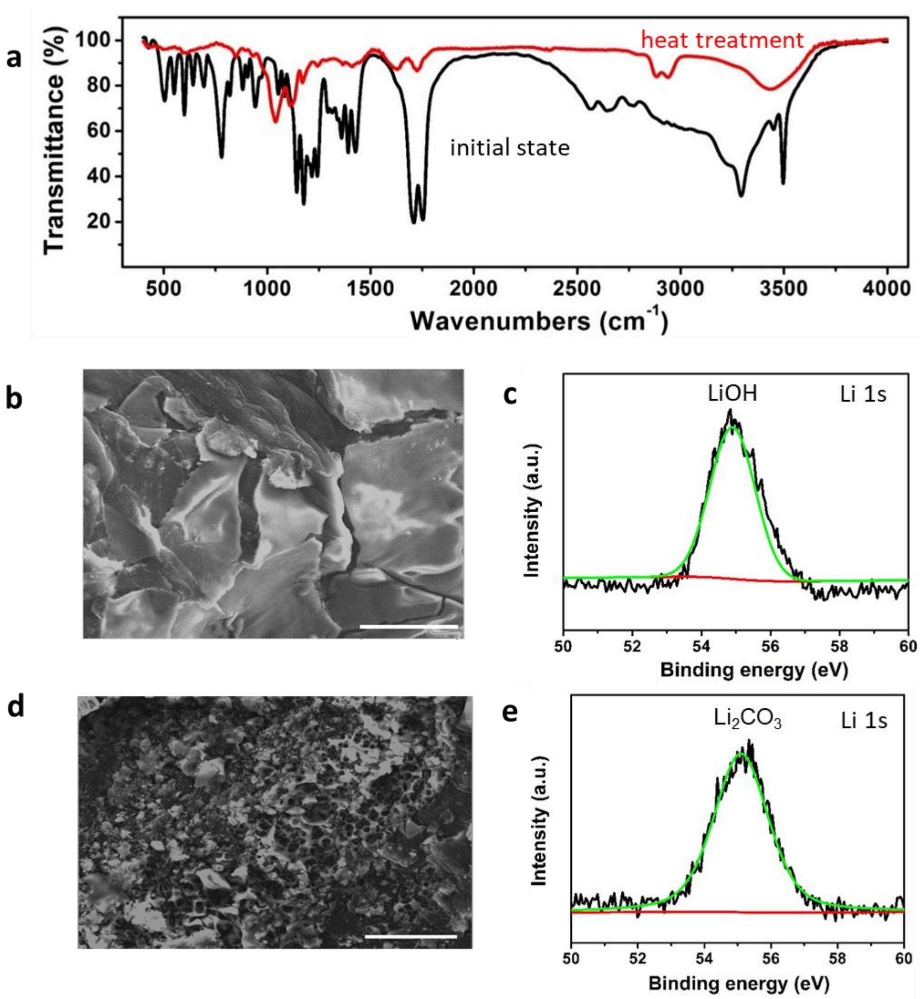

Supplementary Figure 6 | Characterizations of reaction mechanism between molten Li and citric acid. a, IR of citric acid before and after the heat treatment. The disappeared peaks (500~800 cm^{-1}, 1150 cm^{-1}, 1400 cm^{-1}) indicated the decomposition of citric acid after heat treatment, and peaks at 2900 cm^{-1} confirmed the existence of methyl and methylene groups after the heat treatment. **b**, Morphology of the bottom layer of ultrathin Li after the layer was peeled off from planar Cu. **c**, XPS spectra of Li 1s on the bottom ultrathin Li layer. **d**, Morphology of bottom surface of the ultrathin Li after the carbon enriched layer was removed by DOL. The morphology change confirmed the successful removal of the carbon enriched layer. **e**, XPS spectra of Li 1s after the carbon enriched layer was removed by DOL. Similarly, after removal of carbon-enriched layer, the Li 1s peak move to higher binding energy (55.1 eV), indicating the removal of the LiOH on the interphase. Scale bars are 50 μm in b and d.

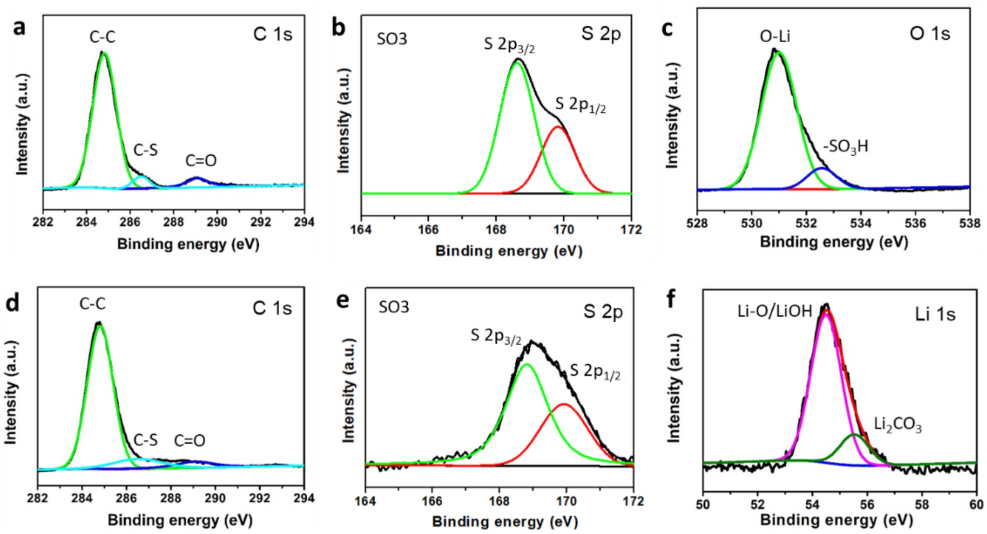

Supplementary Figure 7 | XPS spectra of the bottom surface of ultrathin Li when 2-Naphthalenesulfonic acid was used to improve the wettability. XPS spectra of C 1s (**a**) and S 2p (**b**) before the reaction. XPS spectra of O 1s (**c**), C 1s (**d**), S 2p (**e**), and Li 1s (**f**) after the reaction. The S 2p spectrum exhibits a doublet due to spin-orbit splitting at the binding energies of 168.6 and 169.8 eV for the $2p_{3/2}$ and $2p_{1/2}$ levels and with an area ratio of 2. The binding energy of S 2p on the bottom of ultrathin Li is higher than the one in pure 2-Naphthalenesulfonic acid, confirming the changed environment for S atoms after the heat treatment, which might be attributable to the hydroxyl cleavage. The peak at 54.5 eV can be attributed to Li-O or LiOH.

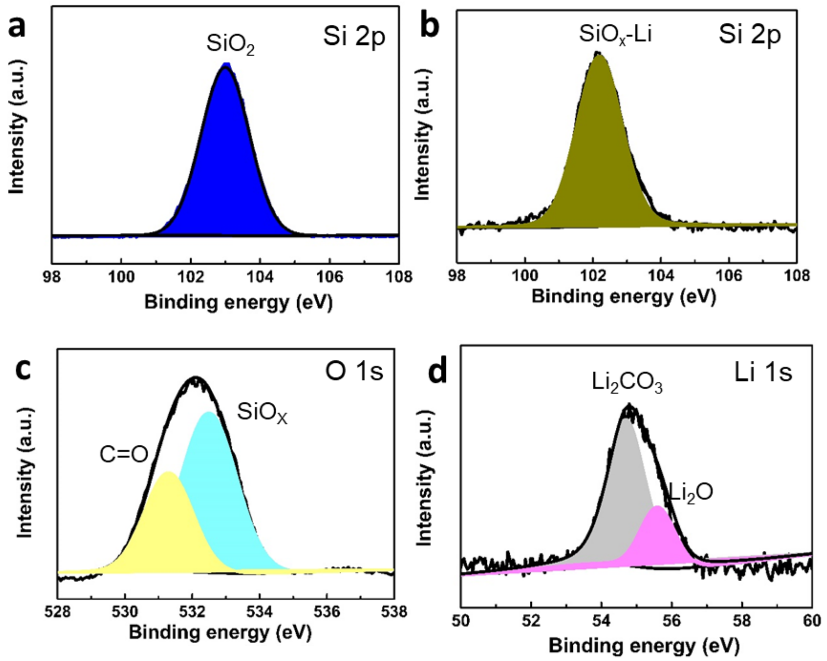

Supplementary Figure 8 | Characterization of new bonds forming on ultrathin Li via TEOS coating. XPS spectra of Si 2p before **(a)** and after **(b)** the reaction. XPS spectra of O 1s **(c)** and Li 1s **(d)** after the reaction of molten Li and TEOS. The fitted peak of SiO_x-Li indicates the new bond forming.

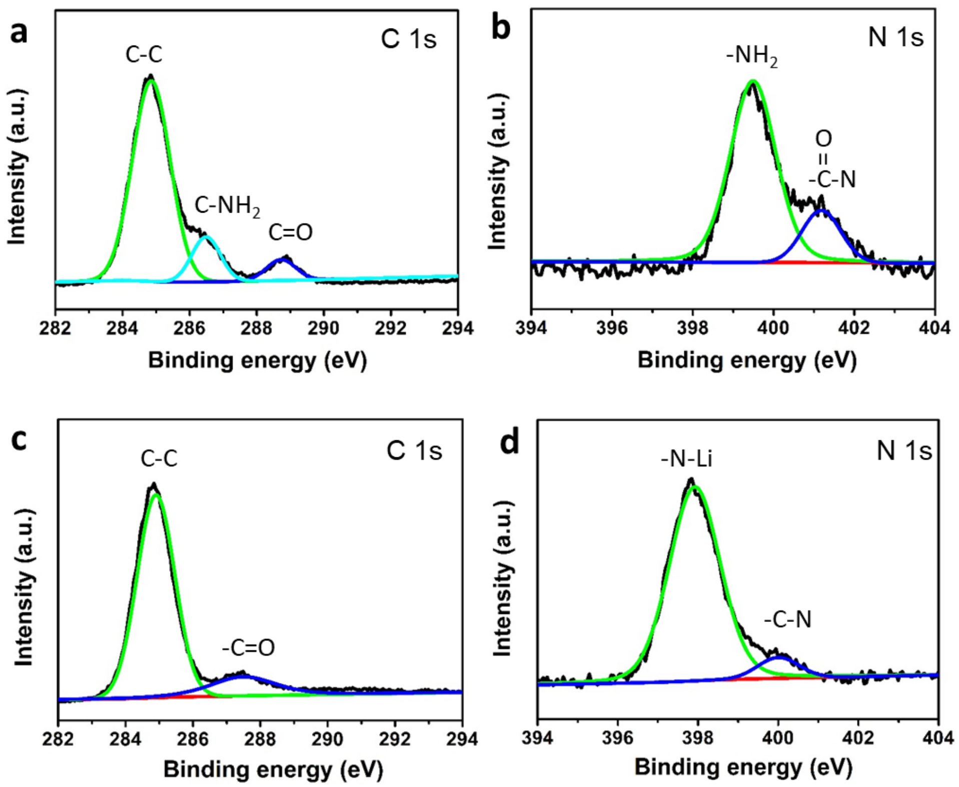

Supplementary Figure 9 | XPS spectra of the bottom surface of ultrathin Li when benzamide was used to improve the wettability. a and **b**, XPS spectra of C 1s (**a**) and N 1s (**b**) before the reaction. **c** and **d**, XPS spectra of C 1s (**c**) and N 1s (**d**) after the reaction.

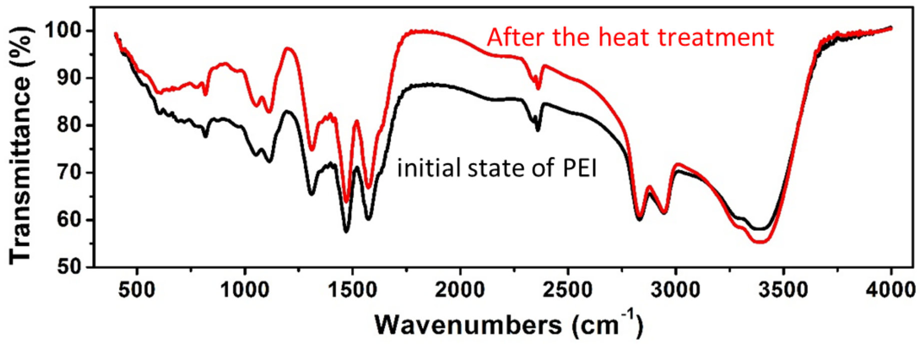

Supplementary Figure 10 | IR of PEI before and after the heat treatment. The unchanged IR spectrum confirms the good thermal stability of PEI at 300 °C.

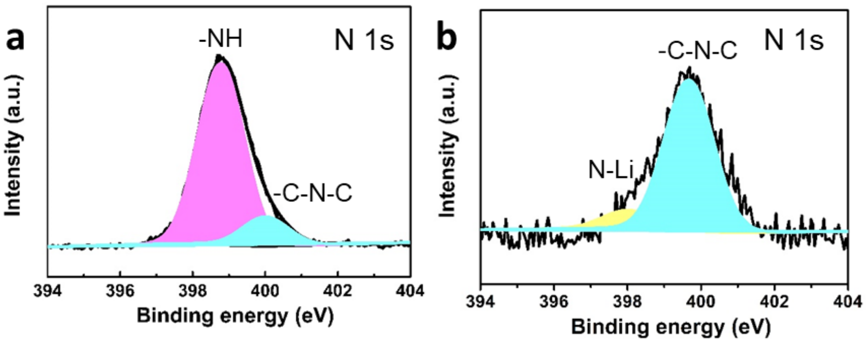

Supplementary Figure 11 | Characterization of the newly formed bonds when PEI was used as a lithiophilic coating. XPS spectra of N 1s before (a) and after (b) the reaction of molten Li with PEI.

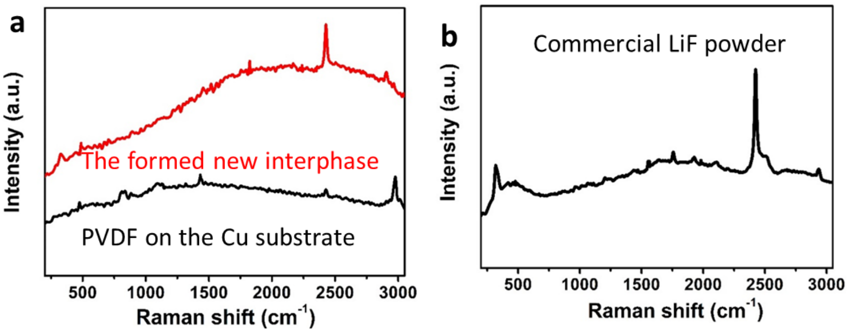

Supplementary Figure 12 | Characterization of the newly formed Li-F between PVDF and molten Li. a, Raman spectra of the new interphase. **b**, Raman spectra of commercial LiF powder.

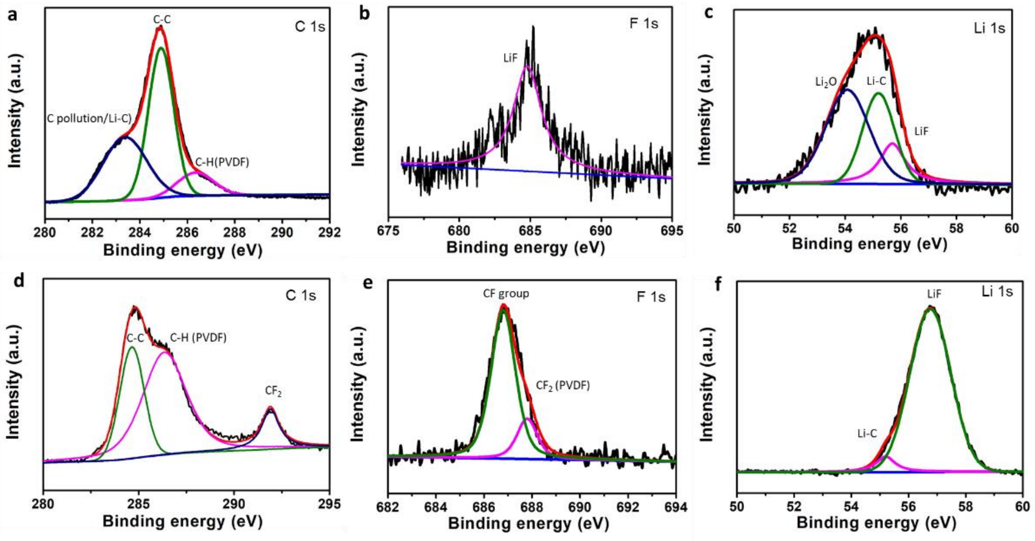

Supplementary Figure 13 | Characterization of the newly formed bonds when PVDF was used as a lithiophilic coating. a and **b**, Li 1s spectra for the original surface (a) and after etching 20 nm by Ar+ sputtering (b). **c** and **d**, F1s spectra for the original surface (c) and after etching 20 nm by Ar+ sputtering (d). **e** and **f**, Li 1s spectra for the original surface (e) and after etching 20 nm by Ar+ sputtering (f).

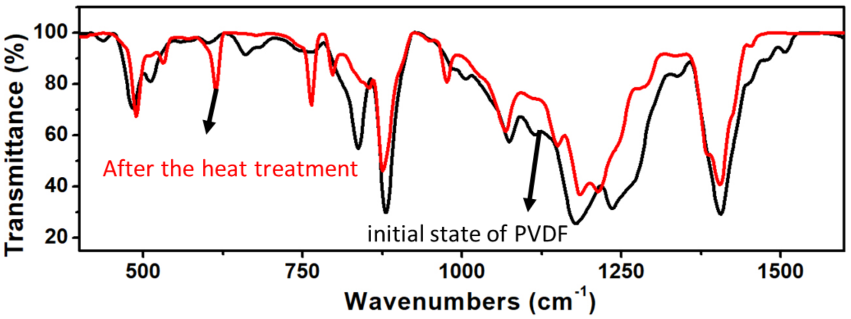

Supplementary Figure 14 | FTIR of PVDF before and after the heat treatment. The disappeared peak at 659 cm^{-1} represents the O=C-N group of NMP, and the disappeared characteristic peak of PVDF at 837 cm^{-1} indicated the changed structure of β-phase PVDF at 300 ºC. Peaks at 613 and 762 cm^{-1} confirmed the existence of α-phase PVDF after the heat treatment.

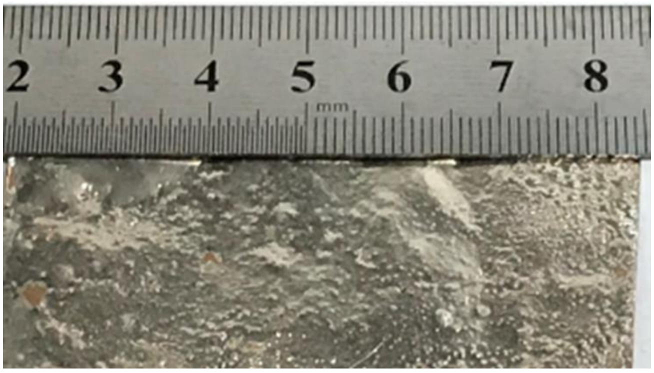

Supplementary Figure 15 | Digital images of ultrathin Li on planar copper.

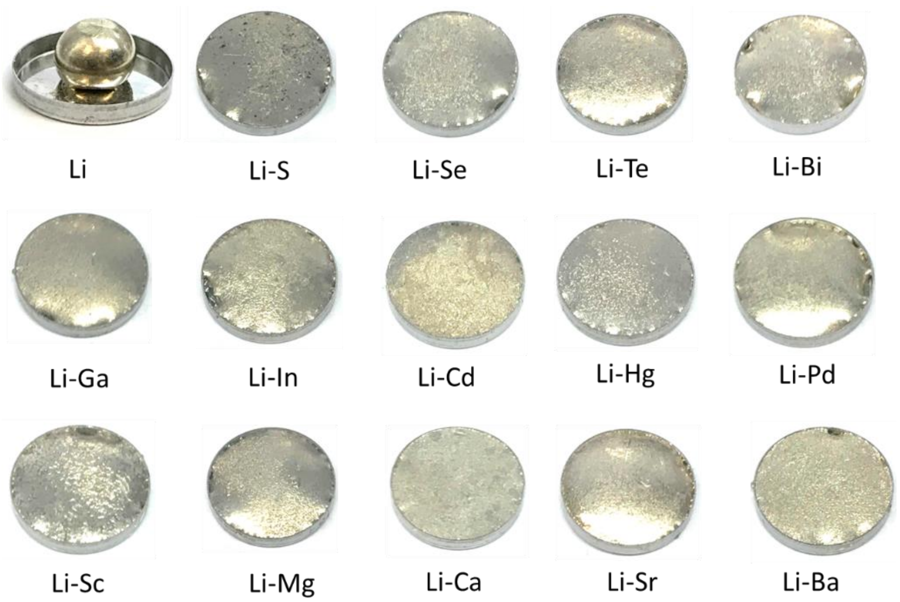

Supplementary Figure 16 | Decreased surface tension of molten Li by adding various elements.

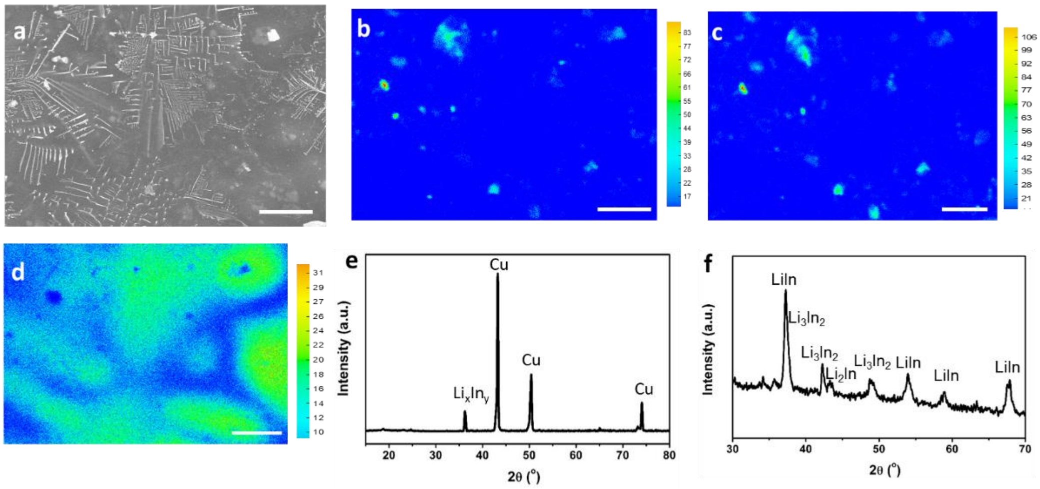

Supplementary Figure 17 | Molten Li with adding In additives directly spread on Cu substrates. **a**, Surface morphology of ultrathin Li_xIn_y (50 wt% In) by EPMA. **b**, Distribution of C on the surface. **c**, Distribution of O on the surface. **d**, Distribution of In on the surface. **e**, XRD pattern of Li_xIn_y (50 wt% In) on the Cu substrate. **f**, XRD pattern of Li_xIn_y without the Cu substrate. The strongest peak shift to 37.28 ^{o}, confirming the formation of LiIn alloy (00-009-0066). However, there are also some peaks for Li_3In_2 (00-033-0616) and Li_2In (03-065-2198). Both the EPMA and XRD result identified the existence of In in the prepared ultrathin anodes. Scale bars are 20 μm in a-d.

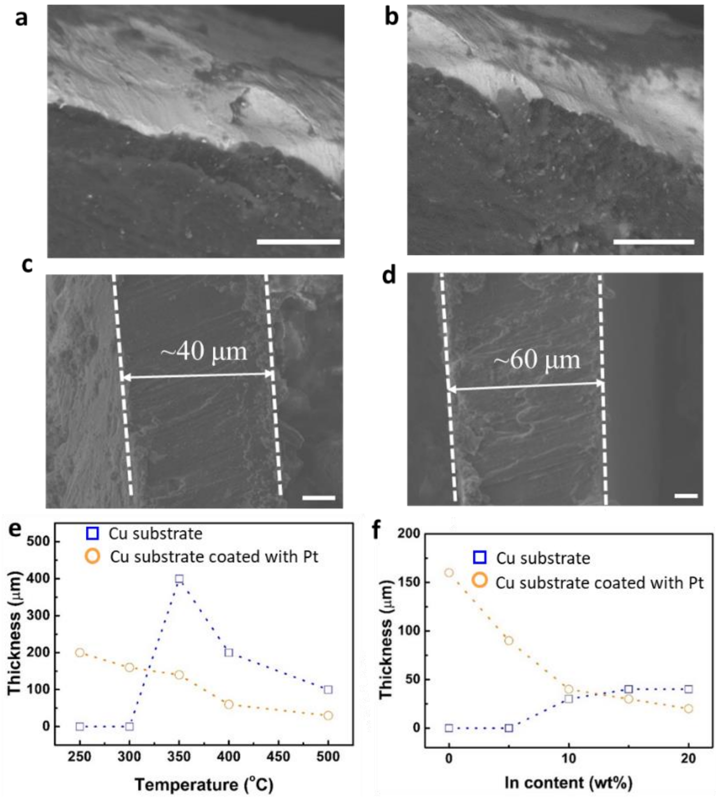

Supplementary Figure 18 | SEM images of ultrathin Li and various parameters that determine the thickness. a-b, SEM images of ultrathin Li prepared via PVDF. The thickness of ultrathin Li when the contacting time was controlled at 2s (**a**) and 5s (**b**). **c-d**, SEM images of ultrathin Li prepared on 20 μm Cu substrate. **e**, The thickness of prepared lithium at different temperatures. **f**, The thickness of prepared lithium when the metal liquids with different compositions. Scale bars are 10 μm in a-d.

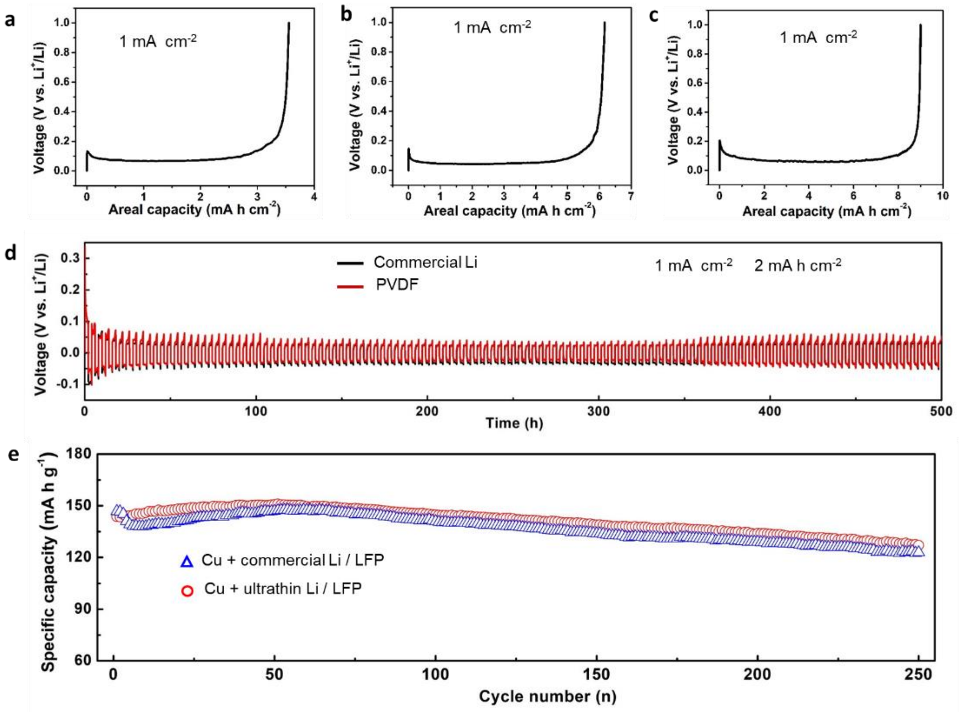

Supplementary Figure 19 | Electrochemical characterization of the ultrathin Li electrodes prepared by PVDF. a-c, Li stripping curves of the ultrathin Li to 1 V versus Li^+/Li with different thickness. **d**, Galvanostatic cycling performance of symmetric cells. The voltage hysteresis of Li plating/stripping on the planar Cu substrate is stable even after cycling for 500 h. The non-square wave behavior for Li striping/plating mainly derives from the dendrites and pits, which is normally observed for Li metal anodes in liquid electrolytes. **e**, Cycling stability of full cells at 0.5 C, respectively. This confirmed that the prepared ultrathin Li has the considerable electrochemical properties with commercial Li anodes.

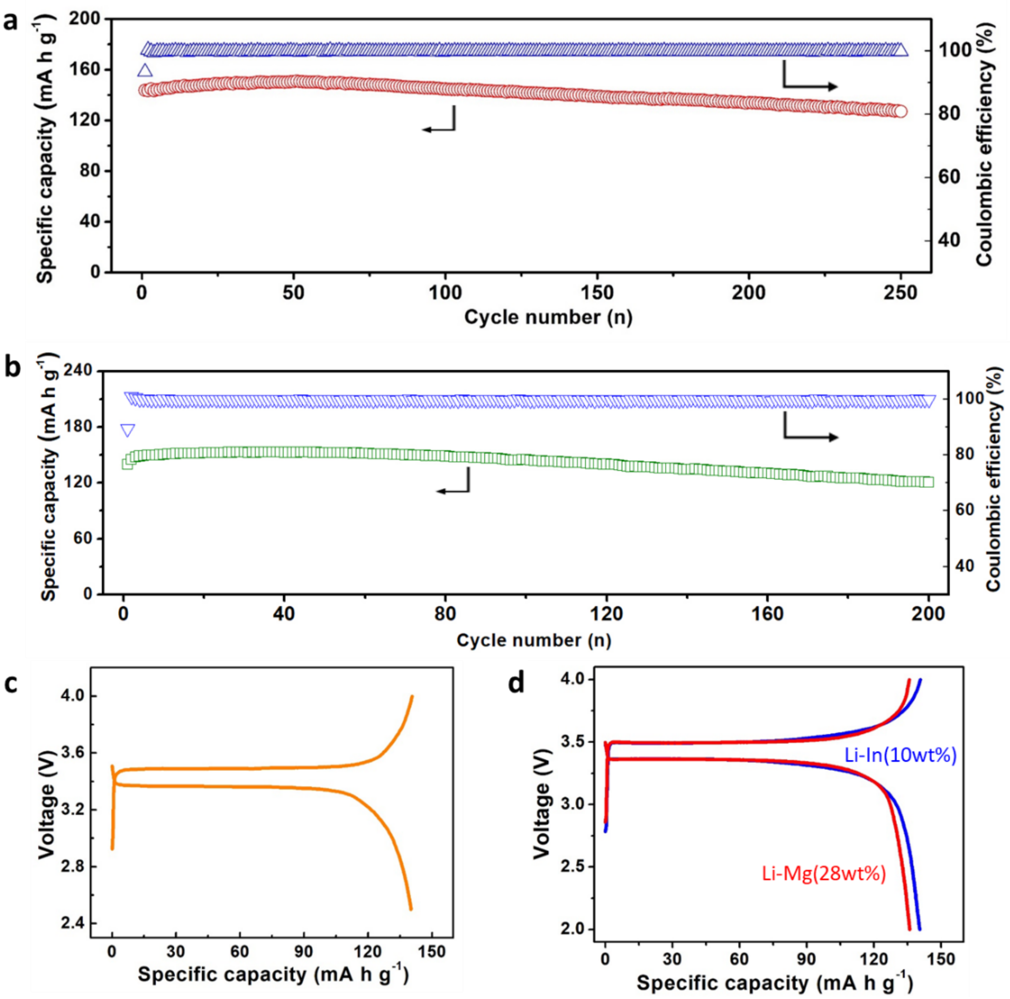

Supplementary Figure 20 | Electrochemical performance of Li/$LiFePO_4$ cells with different anodes, and 1M LiTFSI in DOL/DME with 1 wt% $LiNO_3$ as electrolytes. **a**, Cycling stability of the full cell at 0.5 C with ultrathin Li (30 μm) on planar Cu as the anode. The ultrathin Li anodes were prepared via abietic resin coating. **b**, Cycling stability of the full cell at 0.5 C with ultrathin Li-In (In 10 wt%) on planar Cu as the anode. **c**, Charge/discharge profiles of full cell (Li-In/ $LiFePO_4$) at a cycling rate of 0.5 C. **d**, Charge/discharge profiles of full cells (Li-In/ $LiFePO_4$, Li-Mg/$LiFePO_4$) at a cycling rate of 0.5 C.

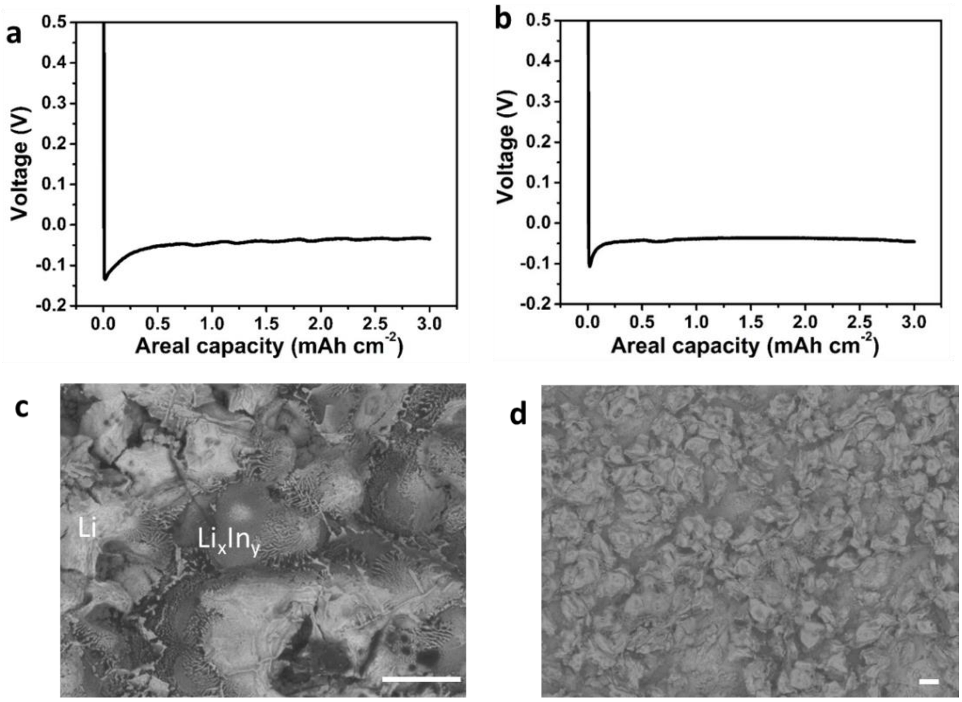

Supplementary Figure 21 | Deposition behaviors of Li metal on planar Cu with a capacity of 3 mA h cm^{-2} at a current density of 1 mA cm^{-2}. **a**, Experimental voltage profile of Li deposition on planar Cu when Li was used as counter electrode. **b**, Experimental voltage profile of Li deposition on planar Cu when Li_xIn_y used as counter electrode. **c**, Backscattered electron image of Li_xIn_y anode. **d**, Backscattered electron image of Li_xIn_y anode after the stripping. Scale bars are 15 μm in c, d.

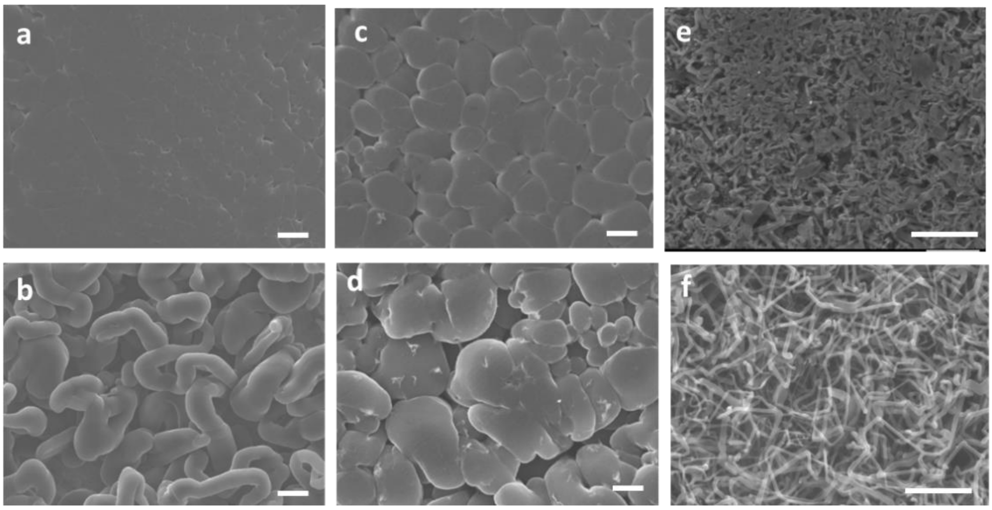

Supplementary Figure 22 | SEM images of Li-Mg (a, c, e) and commercial Li (b, d, f) deposited on the Cu substrates at different current densities. a,**b**, The electrolyte is 1 M LiTFSI in DOL/DME, and the current density is 0.1 mA cm^{-2} with a capacity of 3 mA h cm^{-2}. **c**,**d**, The electrolyte is 1 M LiTFSI in DOL/DME with a current density of 1 mA cm^{-2} and a capacity of 3 mAh cm^{-2}. **e**,**f**, The electrolyte is 1 M $LiPF_6$ in EC/DEC/DMC, and the current density is 1 mA cm^{-2} with a capacity of 3 mA h cm^{-2}. Li-Mg anode showed compacter electrodeposition morphologies than commercial Li anode. At the same time, commercial Li anode showed serious dendrite growth in both ester electrolyte and ether electrolyte. Scale bars are 10 μm in a-f.

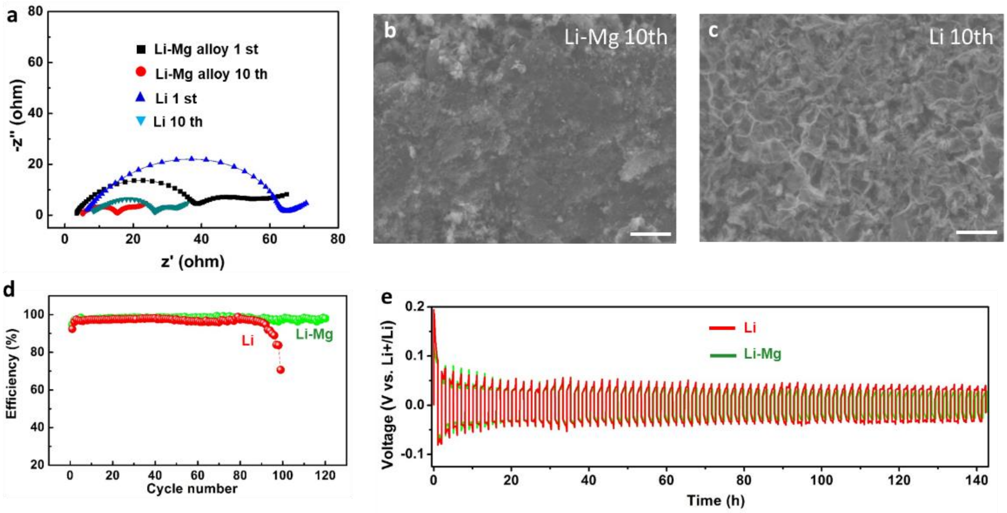

Supplementary Figure 23 | Deposition behaviors of Li and Li-Mg anodes. a, Comparison of resistances of different lithium anodes deposited on planar Cu at different cycles. SEM images of Li-Mg (**b**) and Li (**c**) deposited on Cu substrates after 10 cycles. **d**, Cycling performance of the ultrathin Li and Li-Mg both with thickness of 12 μm on planar Cu substrates at a current density of 1 mA cm^{-2} with a stripping/plating capacity of 1 mA h cm^{-2}. **e**, Galvanostatic cycling of a symmetric cell in the first 140 h. The current density was fixed at 1 mA cm^{-2} with a stripping/plating capacity of 1 mA h cm^{-2}. The ultrathin Li on planar Cu was prepared via abietic resin. Fig. S29d shows that the CE of the cell with Li-Mg anode was stable within 100 cycles. For ultrathin Li on planar Cu, the CE slightly decreased after 90 cycles. Furthermore, the cell with Li-Mg anode showed a low voltage hysteresis of ~40 mV after cycling for 140 h. These results suggest that Li-Mg anode delivers much better electrochemical properties than that of ultrathin Li on planar Cu. Scale bars are 10 μm in b, c.

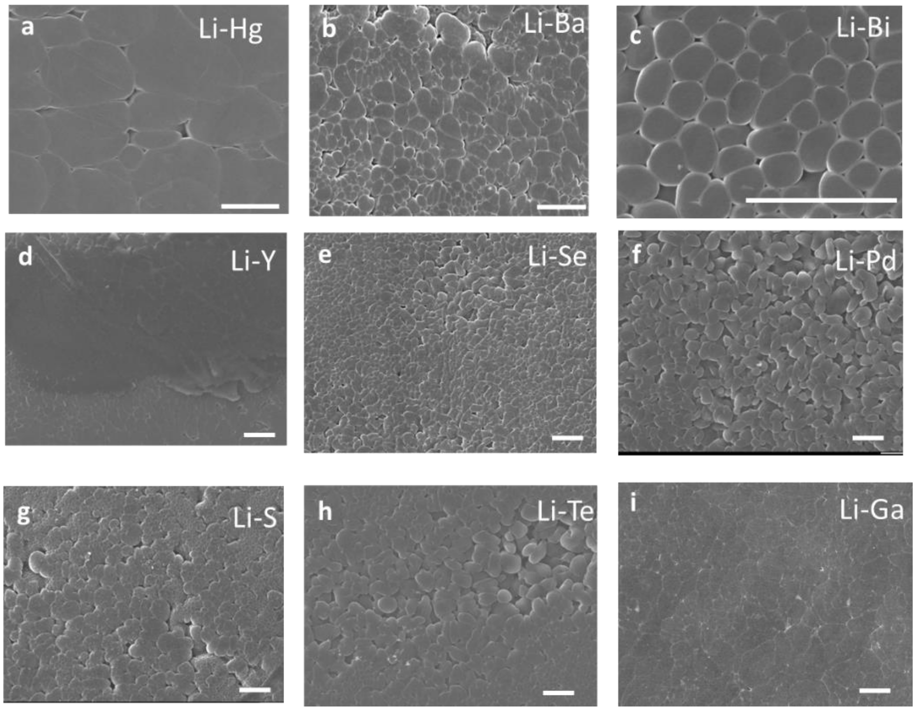

Supplementary Figure 24 | SEM images of Li anodes deposited on Cu substrates. **a**, Li-Hg. **b**, Li-Ba. **c**, Li-Bi. **d**, Li-Y. **e**, Li-SE. **f**, Li-Pd. **g**, Li-S. **h**. Li-Te. **i**. Li-Ga. These ultrathin Li through adding additives exhibit a remarkably compact and uniform electrodeposition, which could be used as anodes in lithium batteries. Scale bars are 10 µm in a-i.

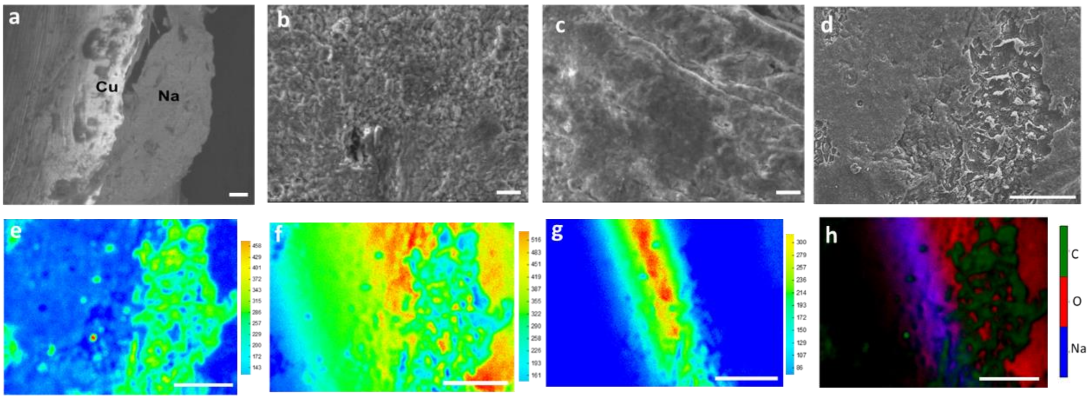

Supplementary Figure 25 | EPMA of ultrathin Na layer when lactic acid was used as the coating. **a**, Thickness of the ultrathin Na layer **b**,**c**, Top surface morphology. **d**, Bottom surface morphology. **e**, Distribution of C on the surface. **f**, Distribution of O on the surface. **g**, Distribution of Na on the surface. **h**, Distribution of C, O, Na on the surface. Scale bars are 10 µm in a-c, and 200 µm in d-h.

Supplementary Tables

Supplementary Table 1 | Physical properties of various organic coatings with a functional group of –COOH.

Name	Formula	Functional group	Melting point	Boiling point	Wettability
Acetic acid	CH_3CO_2H	-COOH	16.7	118	×
Propionic acid	$CH_3CH_2CO_2H$	-COOH	−20.5	141.1	×
Butyric acid	$CH_3CH_2CH_2COOH$	-COOH	−5.3	163.5	×
Hexanoic acid	$CH_3(CH_2)_4CO_2H$	-COOH	−3	205	×
Benzoic acid	C_6H_5COOH	-COOH	122.4	249	√
Oxalic acid	HO_2CCO_2H	-COOH	190	-	√
Adipic acid	$HO_2C(CH_2)_4CO_2H$	-COOH	152-154	337.5	√
Aminoacetic acid	$C_2H_5NO_2$	-COOH<br>-NH_2	182	-	×
Phthalic acid	$C_8H_4O_4$	-COOH	227	-	√
1,3-Benzenedicarboxylic acid	$C_6H_4(COOH)_2$	-COOH	345-348	-	×
Fumaric acid	$HO_2CCH=CHCO_2H$	-COOH	299-300	-	×
Citric acid	$HOOCCH_2C(OH)(COOH)$	-COOH<br>-OH	154	175	√
Abietic acid	$C_{20}H_{29}O_2$	-COOH	172-175	-	√
DL-Lactic acid	$CH_3CH(OH)CO_2H$	-COOH	16.8	122	√
(S)-(+)-Glutamic acid	$HO_2CCH_2CH_2CH(NH_2)CO_2H$	-COOH<br>-NH_2	247	200	√
Methyl 2-aminobenzoate	$H_2NC_6H_4CO_2CH_3$	-COOH<br>-NH_2	24	256	√
meso-2,3-Dibromosuccinic acid	$C_4H_4Br_2O_4$	-COOH<br>-Br	275	-	√

Supplementary Table 2 | Physical properties of various organic coatings with functional groups besides –COOH that improve the wettability of molten Li.

Name	Functional group	Melting point, °C	Concentration	Wettability
Vitamin C	-OH	190-192	5 wt%	√
Polyvinyl alcohol	-OH	230	5 wt%	√
Polyethylene oxide	-OH	87-140	5 wt%	√
Phenolic Resin[1]	-OH	-	5 wt%	√
Polyethylene glycol	-OH	64-66	5 wt%	√
Glucose	-OH	146	5 wt%	√
Polyvinyl pyrrolidone	-N	130	5 wt%	√
Poly(ethylenimine)	-NH	59-60	0.5%	√
Benzamide	$-NH_2$	133	5 wt%	√
β-naphthalene sulfonic acid	$-SO_3H$	124	5 wt%	√
Poly(vinylidene fluoride)	$-CH_2-CF_2$	172	3 wt%	√
Poly(vinyl chloride)	$-CH_2-CHCl$	-	5 wt%	√
Hydrazine Monohydrobromide	H_5BrN_2		5 wt%	√
Trimethylsulfoxonium iodide	$[(CH_3)_3S(O)]I$	169	5 wt%	√
Polyphosphoic Acid	$H_6P_4O_{13}$	16	-	√
Ethylsilicate	Si-O	−77	-	√

Supplementary Table 3 | Data for several possible reactions that could change the wettability of molten Li.

**Possible reactions of molten Li with other chemicals**	**ΔG (298.15 K) kJ mol^{-1}**	**ΔG (523.15 K) kJ mol^{-1}**	**New bonds**	**Bond energy* kJ mol^{-1}**
$2Li+2H_2O=2\underline{LiOH}+H_2$	− 403.6	−407.7	Li-O	427
$Li_2O+H_2O=2\underline{LiOH}$	−79.0	−74.6	Li-O	427
$2Li+2HF=2\underline{LiF}+H_2$	−629.0	−583.1	Li-F	577
$Li_2O+2HF=2\underline{LiF}+H_2O$	−304.3	−250.0	Li-F	577
$LiOH+HF=\underline{LiF}+H_2O$	−112.7	−87.7	Li-F	577
$Li+2HCl=\underline{LiCl}+H_2$	−578.1	−537.0	Li-Cl	469±13
Li2O+HCl=$\underline{LiCl}+H_2O$	−253.47	−203.9	Li-Cl	469±13
$LiOH+HCl=\underline{LiCl}+H_2O$	−87.3	−64.6	Li-Cl	469±13
$Li+HBr=\underline{LiBr}+H_2$	−577.1	−537.3	Li-Br	423
$Li_2O+HBr=\underline{LiBr}+H_2O$	−252.4	−204.3	Li-Br	423
$LiOH+HBr=\underline{LiBr}+H_2O$	−86.7	−64.8	Li-Br	423
$Li+HI=\underline{LiI}+H_2$	−544.0	−506.3	Li-I	352
Li2O+HI=$\underline{LiI}+H_2O$	−219.3	−173.2	Li-I	352
$LiOH+HI=\underline{LiI}+H_2O$	−70.2	−49.3	Li-I	352
$4Li+O_2=2\underline{Li_2O}$	−1123.6	−1066.9	Li-O	341
$2LiOH+CO_2=\underline{Li_2CO_3}+H_2O$	−97.0	−65.6	-	
$6Li+N_2=2\underline{Li_3N}$	−256.0	−202.2	-	-
$Li+PPA\rightarrow\underline{Li_3PO_4}$	<0	<0	-	-
$41Li+5SiO_2=\underline{Li_{21}Si_5}+10Li_2O$	>0	<0	Li-Si	149
$31Li+5SiO=\underline{Li_{21}Si_5}+5Li_2O$	>0	<0	Li-Si	149
$22Li+5Si=\underline{Li_{22}Si_5}$ (alloy)	>0	<0	Li-Si	149

* Bond energies for chemicals refer to the compounds containing element Li after the reactions. The required temperature for reactions were calculated based on the data in thermodynamics database (such as John A. Dean. Handbook of chemistry. Fifteenth Edition (1972)).

Supplementary Table 4 | Combination reaction mechanism to improve the wettability of molten Li at the temperature range of 180~300°C.

Reaction of Li with other chemicals	ΔG (298.15 K) kJ•mol^{-1}	ΔG (523.15 K) kJ•mol^{-1}	Improved wettability
Ge	>0	<0	√Supplementary Reference 2
C, Sn	>0	<0	√Supplementary Reference 3,9
Al_2O_3	>0	<0	√Supplementary Reference 4
ZnO	>0	<0	√Supplementary Reference 5
Mg	>0	<0	√Supplementary Reference 6
Si	>0	<0	√Supplementary Reference 7
Mg, Ca, Sr, Ba	>0	<0	√ This work
Sc, Y	>0	<0	√ This work
Rh, Ir	>0	<0	√ This work
Pd, Pt	>0	<0	√ This work
Ag, Au	>0	<0	√ This work
Cd, Hg	>0	<0	√ This work
Ga, In, Tl	>0	<0	√ This work
Ge, Sn, Pb	>0	<0	√ This work
As, Sb, Bi	>0	<0	√ This work
S, Se, Te	>0	<0	√ This work
Be	>0	>0	× This work
Ti, Zr, Hf	>0	>0	× This work
V, Nb, Ta	>0	>0	× This work
Cr, Mo, W	>0	>0	× This work
Mn, Tc, Re	>0	>0	× This work
Fe, Ru, Os	>0	>0	× This work
Co	>0	>0	× This work
Ni	>0	>0	× This work
Cu	>0	>0	× This work
B	>0	>0	× This work

The reactions could be conducted by the phase diagram[9-21].

Supplementary Table 5 | Energy density of various Li metal batteries.

Different Anodes	LFP Mass mg	LFP Capacity mAh	Li Mass mg	Additives Mass mg	Anode capacity mAh	Anode thickness μm	Energy density Wh kg^{-1}
Commercial Li	2	0.28	23.05	0	110	550	38
Commercial Li	2	0.28	2.10	0	10	50	232.2
Ultrathin Li	2	0.28	0.42	0	2	10	393.3
Ultrathin Li	2	0.28	0.84	0	4	20	335.2
Ultrathin Li	2	0.28	1.26	0	6	30	292
Ultrathin Li	3.6	0.5	1.26	0	6	30	349.8
Ultrathin Li	2	0.28	1.68	0	8	40	258.7
90Li-10In	2	0.28	1.51	0.17	7.19	~36.2	258.7
90Li-50In	2	0.28	0.84	0.84	4	~21.5	258.7

The capacity was calculated based on the (cathode+anode) mass, the average voltage is about 3.4V, thus the corresponding energy density was calculated by (capacity*Voltage)

Supplementary Methods

The X-ray diffraction (XRD) pattern of the ultrathin Li was obtained using an X-ray diffractometer (PANalytical) with Cu Kα radiation (λ=1.54056 Å). Tof-SIMS (TOF. SIMS5 ION-TOF GmbH) was used to obtain elemental distribution and depth profiles of interphase on the ultrathin Li. XPS was conducted on the ESCALab 250Xi (Thermo Scientific) using 200 W monochromatized Al Kα radiation. A device was used to protect Li from oxidation during the sample transfer.

The morphology of ultrathin Li was observed using field emission SEM (JEOL 6701 F). The roughness of the functional coating was measured by AFM (Bruker Multimode 8 with a Nanoscope V controller).

Electrochemistry. Coin-type cells (2032) were assembled in the Ar-filled glovebox (O_2 and H_2O<0.1ppm). Li-stripping curve of the ultrathin Li was measured to obtain the capacity of ultrathin Li. The electrolyte was 1M LiTFSI in DOL/DME with 1wt% $LiNO_3$. Symmetric cells were employed to evaluate the performance of prepared ultrathin Li compared with commercial Li with a current density of 1 mA cm^{-2}. For the long-term galvanostatic discharge/charge test, ultrathin Li (~30 μm) was first plating onto the current collector at 0.5 mA cm^{-2} until the voltage was up to 1 V versus Li^+/Li. The cell was then discharged and charged at 1 mA cm^{-2} with a capacity of 2 mA h cm^{-2}. $LiFePO_4$ was employed as cathode material in full cells with ultrathin Li anodes (10~40 μm). The cathode mass was about 2~3.6 mg. The electrolyte was 1M LiTFSI in DOL/DME with 1 wt% $LiNO_3$, and approximately 50 μL of electrolyte was used in each full cell.

Supplementary References

1. Kajita H. & Imamura Y. Dean. Improvement of physical and biological properties of particleboards by impregnation with phenolic resin. *Wood Sci. Technol.* **26,** 63-70 (1991).

2. Luo, W. *et al.* Transition from Superlithiophobicity to Superlithiophilicity of Garnet Solid-State Electrolyte. *J. Am. Chem. Soc.* **138,** 12258-12262 (2016).

3. Wang, C. *et al.* Universal Soldering of Lithium and Sodium Alloys on Various Substrates for Batteries. *Adv. Energy Mater.* **8,** 1701963 (2018).

4. Han, X. *et al.* Negating interfacial impedance in garnet-based solid-state Li metal batteries. *Nat. Mater.* **16,** 572-579 (2017).

5. Wang, C. *et al.* Conformal, Nanoscale ZnO Surface Modification of Garnet-Based Solid-State Electrolyte for Lithium Metal Anodes. *Nano Lett.* **17,** 565-571 (2017).

6. Fu, K. K. *et al.* Transient Behavior of the Metal Interface in Lithium Metal-Garnet Batteries. *Angew Chem. Int. Ed.* **56,** 14942-14947 (2017).

7. Liang, Z. *et al.* Composite lithium metal anode by melt infusion of lithium into a 3D conducting scaffold with lithiophilic coating. *Proc. Natl. Acad. Sci. USA* **113,** 2862-2867 (2016).

8. Duan, J. *et al.* Lithium-Graphite Paste: An Interface Compatible Anode for Solid-State Batteries. *Adv. Mater*. **31**,1807243 (2019).

9. Saint, J. Exploring the Li-Ga room temperature phase diagram and the electrochemical performances of the Li_xGa_y alloys vs. Li. *Solid State Ionics* **176,** 189-197 (2005).

10. Longo, R. C. *et al.* Phase stability of Li-Mn-O oxides as cathode materials for Li-ion batteries: insights from ab initio calculations. *Phys. Chem. Chem. Phys.* **16,** 11218-11227 (2014).

11. Sangster, J. C-Li (Carbon-Lithium) System. *J. Phase Equilib. Diff.* **28,** 561-570 (2007).

12. Pavlyuk, V., Sozanskyi, M., Dmytriv, G., Indris, S. & Ehrenberg, H. Amendment of the Li-Bi Phase Diagram Crystal and Electronic Structure of Li_2Bi. *J. Phase Equilib.*

Diff. **36,** 544-553 (2015).

13. Okamoto, H. Supplemental Literature Review of Binary Phase Diagrams: Ag-Li, Ag-Sn, Be-Pu, C-Mn, C-Si, Ca-Li, Cd-Pu, Cr-Ti, Cr-V, Cu-Li, La-Sc, and Li-Sc. *J. Phase Equilib. Diff.* **38,** 70-81 (2016).

14. Okamoto, H. Li-Zn (Lithium-Zinc). *J. Phase Equilib. Diff.* **33,** 345-345 (2012).

15. Okamoto, H. Cu-Li (Copper-Lithium). *J. Phase Equilib. Diff.* **32,** 172-172 (2011).

16. Okamoto, H. Ba-Li (Barium-Lithium). *J. Phase Equilib. Diff.* **31,** 489-489 (2010).

17. Okamoto, H. Li-Y (Lithium-Yttrium). *J. Phase Equilib. Diff.* **30,** 216-216 (2009).

18. Okamoto, H. Li-Sc (Lithium-Scandium). *J. Phase Equilib. Diff.* **30,** 117-117 (2008).

19. Braga, M. H., Dębski, A. & Gąsior, W. Li–Si phase diagram: Enthalpy of mixing, thermodynamic stability, and coherent assessment. *J. Alloy. Compd.* **616,** 581-593 (2014).

20. Beutl, A., Cupid, D. & Flandorfer, H. The Li-Sb phase diagram part I: New experimental results. *J. Alloy. Compd.* **695,** 1052-1060 (2017).

21. Fedoseeva, Yu. V. *et al.* Charge polarization in partially lithiated single-walled carbon nanotubes. *Phys. Chem. Chem. Phys.* **20,** 22592-22599 (2018).
