# Supplementary material for: Tuning wettability of molten lithium via a chemical strategy for lithium metal anodes
Source: Nat Commun. 2019 Oct 30;10:4930. doi: 10.1038/s41467-019-12938-4 (PMC6821877; doi:10.1038/s41467-019-12938-4)
Supplement: Supplementary file 2 — Description of Additional Supplementary Files [file 41467_2019_12938_MOESM2_ESM.pdf]

Description of Additional Supplementary Files

File Name: Supplementary Movie 1

Description: The molten Li is put in the stainless-steel vessel at the temperature of 193 ^{o}C. Poor lithiophilicity of molten Li results in spherical Li beads rather than thin layers formed onto planar copper. The contact time between the copper foil and the molten Li is about 8 s.

File Name: Supplementary Movie 2

Description: The right side of the copper foil with circular shape is coated with the abietic resin and abietic resin is used as lithiophilic layers. When abietic resin contact with molten Li at the temperature of 213 ^{o}C, a thermal decomposition reaction of abietic resin occurred, which dramatically improves the wettability of molten Li, and consequently forms ultrathin Li onto copper foil where with abietic resin coating. There is no ultrathin Li formed on the left side of the copper foil. The contact time between the copper foil and the molten Li is about 7 s.

File Name: Supplementary Movie 3

Description: The whole surface of copper foil is coated with abietic resin. When copper foil with abietic resin contact with molten Li at the temperature of 223 ^{o}C, ultrathin Li only forms at the contact area in less than 5 seconds. There is no ultrathin Li formed on the right side of the copper foil.

File Name: Supplementary Movie 4

Description: The whole surface of copper foil is coated with abietic resin. When copper foil with abietic resin contact with molten Li at the temperature of 234 ^{o}C, ultrathin Li forms at the contact area. The contact time between the copper foil and the molten Li is about 7 s. Also, when abietic resin contact with molten Li, the thermal decomposition of abietic resin could be observed obviously.
